# Confounder-Adjusted Differentiation of Colorectal Cancer via Dynamic Propagation of Pathway Influence

**DOI:** 10.3390/ijms262010023

**Published:** 2025-10-15

**Authors:** Larissa Margareta Batrancea, Ömer Akgüller, Mehmet Ali Balcı, Gizem Çalıbaşı Koçal, Lucian Gaban

**Affiliations:** 1 Department of Business, Babeş-Bolyai University, 7 Horea Street, 400174 Cluj-Napoca, Romania; larissa.batrancea@ubbcluj.ro; 2 Department of Mathematics, Faculty of Science, Mugla Sitki Kocman University, 48000 Muğla, Turkey; mehmetalibalci@mu.edu.tr; 3 Oncology Department, Institute of Health Sciences, Dokuz Eylul University, 35340 Izmir, Turkey; 4 Translational Oncology Department, Institute of Oncology, Dokuz Eylul University, 35340 Izmir, Turkey; gizem.calibasi@deu.edu.tr; 5 Faculty of Economics, “1 Decembrie 1918” University of Alba Iulia, 510009 Alba Iulia, Romania

**Keywords:** network analysis, colorectal cancer heterogeneity, tumor laterality, systems biology, therapeutic resistance

## Abstract

Colorectal cancer (CRC) exhibits profound molecular heterogeneity between left-sided and right-sided tumors with distinct therapeutic responses that current static genomic analyses incompletely explain. We developed Dynamic Functional Influence Computation (DynaFIC), a computational framework modeling time-resolved signal propagation through biological networks to quantify functional influence beyond static expression levels. Using the GSE39582 dataset comprising 583 primary CRC samples, we performed confounder-adjusted differential expression analysis controlling for microsatellite instability status, *BRAF* mutations, Tumor Node Metastasis (TNM) stage, age, and sex, identifying 105 laterality-associated genes that underwent DynaFIC temporal network analysis. Right-sided tumors exhibited dramatically higher network connectivity density despite fewer nodes, creating distributed vulnerability patterns with *HOXC6* as the dominant regulator, achieving 200-fold influence through network amplification. Left-sided tumors showed compartmentalized, hierarchical organization with *PRAC1* as the primary regulator and predictable expression-influence scaling. Temporal clustering revealed distinct propagation kinetics: right-sided tumors demonstrated rapid signal saturation requiring early intervention, while left-sided tumors exhibited sustained propagation permitting sequential approaches. Stability Volatility Index analysis showed right-sided tumors maintain significantly higher systemic vulnerability. These findings establish anatomical location as a fundamental network organizational principle, suggesting that incorporating temporal dynamics into cancer analysis reveals therapeutically relevant differences for precision medicine applications in colorectal cancer.

## 1. Introduction

Colorectal cancer (CRC) is among the most common malignancies worldwide and accounts for approximately 9% of all cancer-related deaths [1,2]. Beyond its overall prevalence, CRC is increasingly recognized as a heterogeneous disease, with tumor location (left-sided versus right-sided colon) emerging as a critical determinant of clinical outcomes and molecular characteristics. Patients with right-sided CRCs generally have worse prognoses and respond differently to therapy compared to those with left-sided CRCs [3]. For instance, tumors originating in the left colon (especially *RAS* wild-type) derive significantly greater benefit from anti-EGFR monoclonal antibody therapies, whereas right-sided tumors tend to be less responsive—a finding reflected in modern treatment guidelines stratifying therapy by tumor sidedness [4,5]. Underlying these disparities are distinct molecular profiles: right-sided CRCs frequently exhibit high levels of microsatellite instability (MSI) and an enriched prevalence of oncogenic *BRAF* V600E mutations [6], whereas left-sided CRCs are typically chromosomally unstable and carry *APC* and *TP53* tumor-suppressor mutations at higher frequencies [7]. These molecular differences, along with variations in gene expression subtypes and tumor microenvironment composition, underscore the biological divergence between CRC subtypes based on sidedness and partly explain the observed prognosis and therapy response gaps.

Despite such insights, current approaches for analyzing functional perturbations in cancer often fall short of capturing the full complexity of tumor biology. Most transcriptomic analyses rely on static gene-expression snapshots, identifying differentially expressed genes between tumor subsets or conditions without regard for temporal changes [8,9]. Such static analyses ignore the dynamic progression of molecular events and therefore cannot detect transient or sequential dysregulations that occur during tumor development and treatment response. Similarly, network-based analyses in oncology typically treat molecular interaction maps as static, even though cellular signaling networks are inherently dynamic systems [10,11,12,13]. Important context-specific pathway activations or short-lived signaling events may thus remain undetected when using conventional static frameworks [14,15,16,17]. These limitations highlight the need for analytical methods that explicitly incorporate temporal dynamics into cancer systems biology.

We posit that a dynamic view of functional perturbations—examining how molecular influences propagate and evolve over time—can reveal regulatory disruptions that static methods miss. In this context, we introduce the concept of dynamic functional influence, referring to the time-resolved impact that a perturbation (such as a genetic alteration or signaling event) exerts across a biological network. Capturing this dynamic functional influence requires moving beyond traditional steady-state analyses to model the propagation of signals through pathways as a function of time. To achieve this, we developed a novel computational framework designed to compute dynamic functional influence in cancer networks, addressing the gap left by static gene-centric or static network approaches.

Dynamic Functional Influence Computation (DynaFIC) is the proposed framework that implements this time-resolved analysis. DynaFIC models the propagation of signaling activity through biological interaction networks over a defined time course, rather than assuming a static equilibrium. By simulating how signals originating from a perturbed gene or pathway spread, amplify, and attenuate through network connections, DynaFIC can detect transient dysregulations and cascade effects that would be invisible to static gene expression or pathway analyses. In essence, this approach captures the dynamics of functional influence—allowing us to observe not only which pathways are dysregulated in a tumor, but also when and for how long such dysregulations occur. Modeling the timing of signal propagation is particularly crucial in complex diseases like CRC, where the temporal context of pathway activation can critically influence phenotypic outcomes (such as cell proliferation, metastasis, or drug sensitivity).

Moreover, the DynaFIC framework provides quantitative metrics that enable direct comparisons of functional disruptions between left- and right-sided tumors. We define a DynaFIC score for each network node (e.g., a gene or protein), representing the cumulative influence that node exerts across the dynamic network simulation. Additionally, a Stability Volatility Index (SVI) measures the fluctuation of a node’s influence over time—a high SVI indicates transient, burst-like signaling activity, whereas a low SVI denotes a sustained influence. We also introduce a Context Modulation Index (CMI) to gauge how contained a perturbation’s effects are within specific network communities; a higher CMI implies that dynamic influences remain largely confined to particular modules, whereas a lower CMI indicates that a perturbation spreads its effects across multiple modules of the network. Armed with these metrics, and by performing temporal clustering to group similar time-course perturbation patterns, DynaFIC enables a direct side-by-side comparison of left-sided CRC versus right-sided CRC network dysregulation. This approach can pinpoint differences in the timing, magnitude, and modular distribution of signaling perturbations between tumor sides, providing a systems-level view of how left- and right-sided CRCs diverge in their functional network behavior.

Our study leverages DynaFIC to unveil side-specific functional disruptions in colorectal cancer by integrating temporal dynamics into functional influence analysis. By interrogating when and how signaling pathways become aberrantly activated or suppressed in left- versus right-sided tumors, this framework fills a critical gap in computational oncology. Traditional genomic and network analyses lack a temporal dimension, whereas DynaFIC demonstrates that incorporating time-resolved signal propagation can uncover transient vulnerabilities and context-dependent dysregulations that static approaches overlook. This work thus represents a convergence of systems biology and oncology, applying time-resolved network modeling to tumor heterogeneity. In doing so, we provide novel insights into the dynamic regulatory differences between left- and right-sided CRC, with potential implications for more tailored therapeutic strategies and precision medicine in colorectal cancer.

## 2. Results

### 2.1. Distinct Functional Signatures in Left Versus Right-Sided CRC

**Figure 1:**
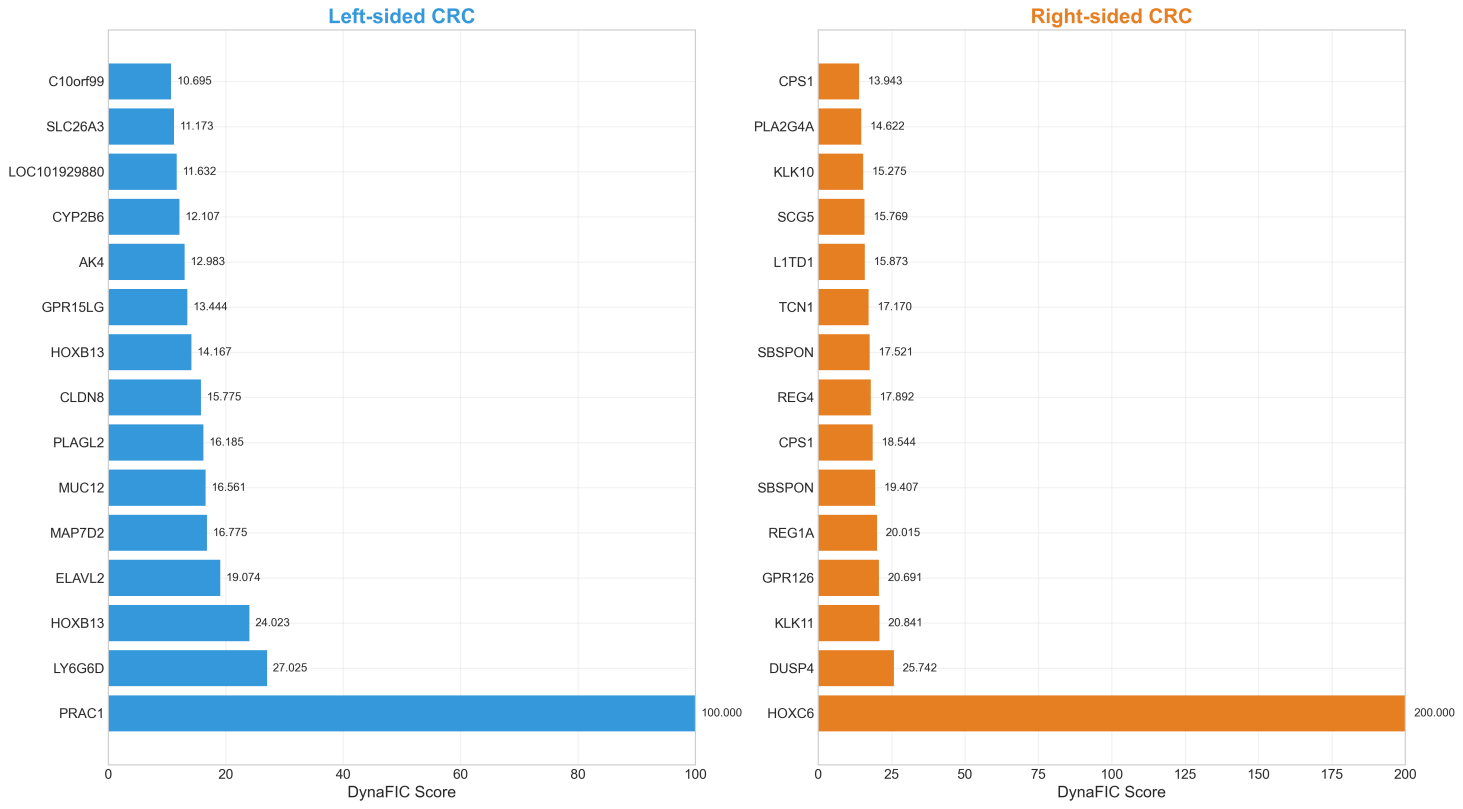
Th top 15 DynaFIC-scored genes reveal distinct molecular signatures between left-sided and right-sided colorectal cancer. Note the different scale ranges between left-sided (0–100) and right-sided (0–200) tumors, indicating greater network influence magnitudes in right-sided disease.

The application of the DynaFIC framework to differentially expressed genes revealed profoundly divergent molecular landscapes between left-sided and right-sided colorectal cancers (Figure 1). The magnitude and identity of top-scoring genes differed dramatically between anatomical locations, with right-sided tumors exhibiting substantially higher DynaFIC scores overall, suggesting more centralized regulatory architectures compared to the distributed networks characteristic of left-sided disease.

Complete rankings and statistical details for the top twenty genes from each anatomical location are provided in Table 1, which demonstrates the consistent elevation of DynaFIC scores in right-sided tumors across multiple functional categories.

In left-sided colorectal cancer, *PRAC1* emerged as the highest-scoring gene with a DynaFIC score of 100.00, despite marked differential expression (log_2_FC = −3.78). This protein-related arginine, lysine, and carboxylic acid transporter, normally involved in amino acid transport and cellular metabolism, represents a critical component of nutrient homeostasis whose downregulation signals disrupted metabolic architecture. The subsequent high-ranking genes—*LY6G6D* and *HOXB13*—further emphasize the predominance of developmental regulatory markers among left-sided influential genes. Notably, multiple secreted factors and transcriptional regulators populated the top twenty genes, including *MUC12*, *PLAGL2*, and the tight junction protein *CLDN8*, collectively painting a picture of disrupted epithelial integrity and secretory functions.

The molecular signature of right-sided tumors contrasted starkly, dominated by genes associated with embryonic patterning, metabolic regulation, and developmental control pathways. *HOXC6* achieved an exceptional DynaFIC score of 200.00—exactly twice that of the highest left-sided gene—despite moderate upregulation (log_2_FC = 1.59). This dramatic network-mediated amplification suggests *HOXC6* occupies a critical regulatory hub position, potentially modulating multiple downstream developmental pathways through its role as a homeobox transcription factor controlling anteroposterior axis specification. The dual specificity phosphatase *DUSP4* ranked second with a score of 25.74, indicating activation of *MAP* kinase regulatory pathways that may confer adaptive advantages under oncogenic stress conditions.

The presence of developmental regulators among right-sided top genes provided compelling evidence for dedifferentiation and embryonic program reactivation. *HOXC6*, along with *HOXB6* and *HOXA10-HOXA9*, represents homeobox transcription factors normally silenced in the adult colon, suggesting reversion to a more primitive cellular state consistent with the midgut embryological origin of the proximal colon. This developmental theme extended to other high-scoring genes, including the regenerating islet-derived proteins *REG1A* and *REG4* and the secreted glycoprotein *SBSPON*. The concurrent elevation of metabolic regulators—*CPS1*, *KLK11*, and *TCN1*—indicates complex biosynthetic programs that may contribute to the metabolically active phenotype characteristic of right-sided tumors.

Comparative analysis of the score distributions revealed systematic differences in network influence patterns. Right-sided tumors demonstrated a power-law-like distribution with a small number of extremely high-scoring hub genes, whereas left-sided tumors showed more uniform score distributions across the top genes. The mean DynaFIC score for the top twenty right-sided genes (29.67 ± 46.12) significantly exceeded that of left-sided genes (19.38 ± 20.95), despite comparable magnitudes of differential expression, indicating more efficient signal propagation through right-sided tumor networks.

### 2.2. Network Influence Heterogeneity Between Tumor Sides

**Figure 2:**
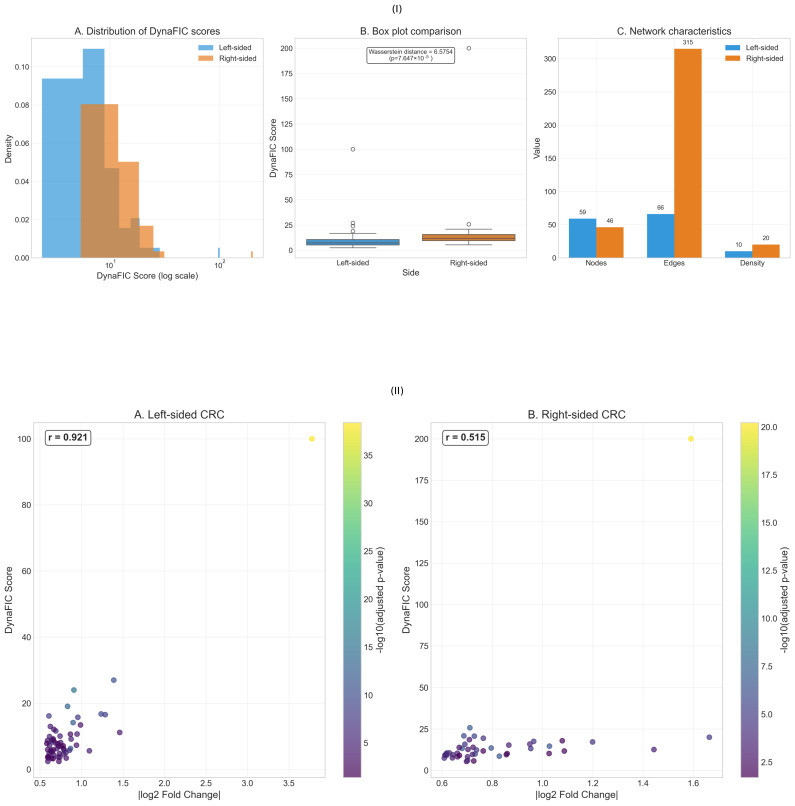
(**I**) Comprehensive analysis of DynaFIC score distributions reveals greater network heterogeneity in right-sided colorectal cancer. (I-A) Distribution comparison on logarithmic scale, (I-B) box plot comparison with statistical significance, (I-C) network structural characteristics comparison. (**II**) Network propagation reveals differential relationships between expression magnitude and functional influence. Scatter plots showing absolute log2 fold change versus DynaFIC scores for left-sided CRC (II-A) and right-sided CRC (II-B). Points colored by statistical significance reveal distinct correlation patterns between tumor sides.

The distribution of DynaFIC scores across all analyzed genes revealed fundamental differences in network organization between left-sided and right-sided colorectal cancers (Figure 2I). The score distributions exhibited distinct topological features that suggest divergent mechanisms of functional signal propagation through the respective tumor networks, with implications for understanding their differential therapeutic vulnerabilities.

Analysis of the DynaFIC score distributions on a logarithmic scale demonstrated that both tumor locations followed heavy-tailed distributions, but with markedly different characteristics (Figure 2I-A). Left-sided tumors displayed a more compressed distribution, with most genes clustering between scores of 1 and 10, exhibiting a sharp peak in the density histogram. In contrast, right-sided tumors showed a broader, more dispersed distribution extending to substantially higher values, with an appreciable fraction of genes achieving scores exceeding 20. This rightward shift and increased dispersion in right-sided tumors suggests the presence of highly influential regulatory hubs that dominate network dynamics.

The box plot comparison further quantified these distributional differences, revealing that while the median DynaFIC scores were comparable between tumor sides, the variance and presence of extreme values differed dramatically (Figure 2I-B). Right-sided tumors harbored numerous outlier genes with DynaFIC scores exceeding 25, including one surpassing 200, whereas left-sided tumors showed a more constrained range with maximum values rarely exceeding 100. The Wasserstein distance of 6.5754 between the two distributions represents a statistically significant shift ($p=7.647\times 10^{-6}$) when considering the logarithmic scale of the measurements.

Network structural analysis provided mechanistic insights into the observed score distributions (Figure 2I-C). Right-sided tumor networks comprised 46 nodes compared to 59 in left-sided tumors, representing a 22% decrease in network size. More strikingly, the edge count revealed a dramatic disparity, with right-sided networks containing 315 edges versus only 66 in left-sided networks—a nearly five-fold difference. This dramatic increase in connectivity translated to a higher network density in right-sided tumors (density = 20 versus 10 in left-sided), indicating more extensive crosstalk between functionally related genes. The denser connectivity pattern in right-sided networks provides multiple alternative paths for signal propagation, potentially explaining both the higher maximum scores and the broader score distribution observed.

The mean DynaFIC score for right-sided genes (16.57 ± 35.42) significantly exceeded that of left-sided genes (10.08 ± 15.89), despite the smaller number of genes analyzed, indicating more efficient signal amplification through right-sided tumor networks. Complete results for all analyzed genes are provided in Appendix A (Table A1 and Table A2).

The relationship between differential expression magnitude and final DynaFIC scores revealed contrasting patterns of network-mediated signal amplification between left-sided and right-sided colorectal cancers (Figure 2II). While both tumor types demonstrated correlations between expression changes and network influence, the strength and characteristics of these relationships differed substantially, underscoring distinct mechanisms of functional signal propagation within their respective regulatory architectures.

In left-sided colorectal cancer, the correlation between absolute log2 fold change and DynaFIC score demonstrated a remarkably strong linear relationship (r = 0.921), indicating that expression magnitude serves as a reliable predictor of network influence in these tumors (Figure 2II-A). Genes spanning the fold change range from 0.5 to 3.8 exhibited a proportional scaling of DynaFIC scores, with the highest-expressing genes consistently achieving the greatest network influence. The color gradient representing statistical significance (−log10 adjusted *p*-value) revealed that highly significant differential expression aligned closely with elevated DynaFIC scores, suggesting that statistically robust expression changes translate efficiently into functional network perturbations. This tight coupling indicates that left-sided tumor networks operate through relatively direct amplification mechanisms, where the magnitude of initial perturbation determines the extent of downstream influence propagation.

Right-sided tumors exhibited a fundamentally different relationship, with a moderate correlation (r = 0.515) between expression magnitude and network influence (Figure 2II-B). Despite the expanded scale of the y-axis necessitated by DynaFIC scores reaching 200, the scatter pattern revealed substantial deviation from linear scaling. Multiple genes with modest fold changes (0.6–1.0) achieved disproportionately high DynaFIC scores, while others with similar expression changes remained confined to low network influence values. *HOXC6*, the highest-scoring gene, exemplified this phenomenon with a DynaFIC score of 200.0 arising from a fold change of only 1.59, representing a nearly 126-fold amplification ratio. The distribution of highly significant genes (indicated by yellow coloring) across various DynaFIC levels, rather than clustering at high scores, demonstrated that statistical significance in differential expression analysis provides limited predictive value for network influence in right-sided tumors.

The contrasting correlation strengths revealed fundamental architectural differences in network organization between tumor sides. Left-sided tumors demonstrated hierarchical signal processing, where expression magnitude determines network position and influence capacity through predictable scaling relationships. The strong positive correlation suggests that these networks maintain proportional response characteristics, enabling reliable signal transmission from initial perturbations to downstream effects. Conversely, right-sided tumors exhibited context-dependent amplification, where network topology and connectivity patterns override expression magnitude in determining functional influence. This architecture creates potential for both signal amplification and dampening independent of initial perturbation strength.

The fold change ranges differed significantly between sides, with left-sided genes spanning 0.5 to 3.8 compared to the more compressed 0.6 to 1.7 range in right-sided tumors. This observation suggests that left-sided cancers achieve functional diversity through varied expression magnitudes, while right-sided tumors rely on network-mediated amplification of relatively modest expression changes. The statistical significance patterns further supported this interpretation, with left-sided tumors showing clear correspondence between significance and influence, while right-sided tumors demonstrated distributed significance levels across the entire DynaFIC spectrum.

### 2.3. Temporal Propagation Patterns

**Figure 3:**
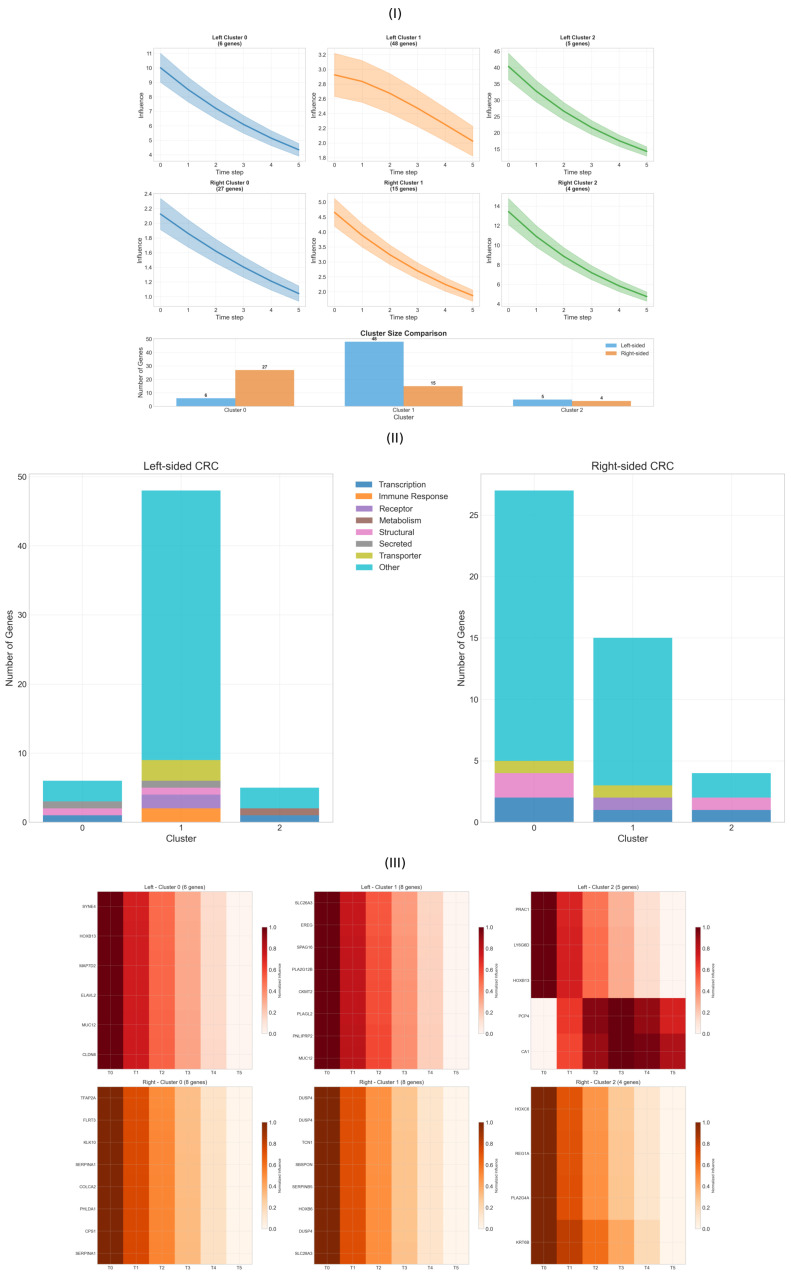
(**I**) Temporal propagation patterns in colorectal cancer subnetworks with outlier-resistant clustering. Dynamic signatures showing three distinct temporal clusters for left-sided and right-sided CRC, with cluster size comparison revealing inverted distribution patterns between tumor types. (**II**) Functional category distribution across temporal clusters showing distinct organizational patterns between left-sided and right-sided CRC. Left-sided tumors display functional homogeneity, while right-sided tumors show more balanced category distributions. (**III**) Temporal dynamics of top genes by cluster showing normalized influence propagation over time T0–T5. All clusters exhibit decay kinetics but with distinct temporal signatures reflecting different regulatory mechanisms.

Network-based influence propagation revealed distinct temporal signatures that differentiate left- and right-sided colorectal cancers through outlier-resistant clustering analysis. Through systematic classification of gene trajectories across diffusion time steps, we identified three characteristic propagation patterns for each tumor type, each associated with specific biological functions and regulatory mechanisms (Figure 3).

Analysis of 21 left-sided and 24 right-sided differentially expressed genes revealed fundamentally different temporal dynamics and cluster size distributions between anatomical locations (Figure 3I). The outlier-resistant clustering approach successfully partitioned genes into three distinct groups based on their propagation profiles, with dramatically different cluster compositions between sides.

Left-sided colorectal cancer demonstrated a highly asymmetric cluster distribution dominated by Cluster 1, containing 48 genes (81% of analyzed genes), while Clusters 0 and 2 comprised only 6 and 5 genes, respectively. Cluster 0 exhibited moderate initial influence that declined steadily over time, including key regulatory genes such as *ELAVL2*, *MAP7D2*, and the mucin *MUC12*, along with the structural protein *CLDN8*. Cluster 1 represented the dominant temporal pattern, characterized by consistent moderate-level influence propagation across all time steps, encompassing diverse functional categories including metabolic enzymes (*PLAGL2*), receptors (*GPR15LG*), and transporters (*SLC26A3*). Cluster 2 displayed the highest initial influence values but followed a similar decay pattern, notably containing *PRAC1* with the maximum DynaFIC score, alongside developmental regulators *LY6G6D* and *HOXB13*.

Right-sided CRC exhibited a markedly different organization with more balanced cluster distributions: Cluster 0 contained 27 genes (59%), Cluster 1 had 15 genes (33%), and Cluster 2 comprised 4 genes (9%). Cluster 0 showed the most gradual decay trajectory and included metabolic regulators (*CPS1*), regeneration factors (*REG4*), and developmental transcription factors (*HOXA10-HOXA9*). Cluster 1 demonstrated a steeper initial decline with intermediate influence levels, enriched for phosphatases (*DUSP4*), kallikreins (*KLK11*), and signaling molecules (*GPR126*, *TCN1*). Cluster 2 contained the highest-influence genes with the steepest decay profiles, dominated by the transcription factor *HOXC6* achieving maximum network influence, along with *REG1A* and *PLA2G4A*.

The inverted cluster size distribution between tumor sides (1>0>2 for left-sided versus 0>1>2 for right-sided) reflects fundamentally different network organization principles, with left-sided tumors showing homogeneous temporal responses and right-sided tumors displaying more stratified influence hierarchies.

The functional composition of clusters revealed systematic differences in biological organization between left- and right-sided CRC (Figure 3II). Left-sided tumors showed functional homogeneity within clusters, with Cluster 1 dominated by “Other” category genes (80%), while Clusters 0 and 2 displayed mixed functional representations including secreted factors, structural proteins, and transcriptional regulators.

Right-sided CRC demonstrated more balanced functional distributions across all clusters, with each cluster containing representatives from multiple functional categories. Cluster 0 showed a predominance of “Other” category genes (80%) but included transcriptional regulators and structural proteins. Cluster 1 maintained similar functional diversity with strong representation of regulatory and signaling molecules. Cluster 2, despite containing only 4 genes, exhibited remarkable functional diversity with transcription factors (*HOXC6*), metabolic enzymes, and structural proteins (*KRT6B*), suggesting coordinated regulation of multiple biological processes through network-mediated amplification.

The predominance of transcription factors across right-sided clusters (*HOXC6*, *HOXA10-HOXA9*, *HOXB6*) aligns with the developmental reprogramming characteristic of proximal colon tumors, while the enrichment of regeneration-associated genes (*REG1A*, *REG4*) reflects the tissue repair pathways activated in right-sided malignancies. Left-sided clusters showed greater enrichment for metabolic and transport functions, consistent with their role in nutrient absorption and processing.

Heatmap visualization of the top genes per cluster revealed the fine-scale temporal dynamics underlying each propagation pattern (Figure 3III). All clusters demonstrated decay-type kinetics but with distinct temporal signatures that reflect different regulatory mechanisms and network positions.

In left-sided Cluster 0, genes like *ELAVL2* and *MAP7D2* showed immediate high response at T = 0 followed by steady exponential decay, indicating direct responsiveness to initial network perturbations. Cluster 1 genes displayed more sustained intermediate-level responses with gradual decline, suggesting integration of multiple network inputs. Cluster 2 exhibited the most dramatic temporal profile, with *PRAC1* showing maximal initial influence followed by sharp decay, characteristic of hub genes that rapidly redistribute network energy.

Right-sided clusters revealed more complex temporal architectures. Cluster 0 genes demonstrated relatively stable decay patterns with moderate initial values, suggesting robust regulatory control mechanisms. Cluster 1 showed intermediate dynamics with a steeper initial decline, indicating a more sensitive response to network perturbations. Cluster 2 displayed the most extreme temporal signature, with *HOXC6* achieving maximum influence at T = 0, followed by the steepest decay profile, consistent with a master regulatory function that rapidly coordinates downstream target activation.

The distinct temporal signatures suggest different regulatory mechanisms: immediate high responders likely represent direct targets of primary perturbations with rapid signal dissipation, sustained moderate responders indicate cascade-mediated effects with buffered decay kinetics, and extreme responders suggest master regulatory hubs that coordinate rapid network-wide responses through concentrated influence redistribution.

### 2.4. Functional Heterogeneity Analysis

**Figure 4:**
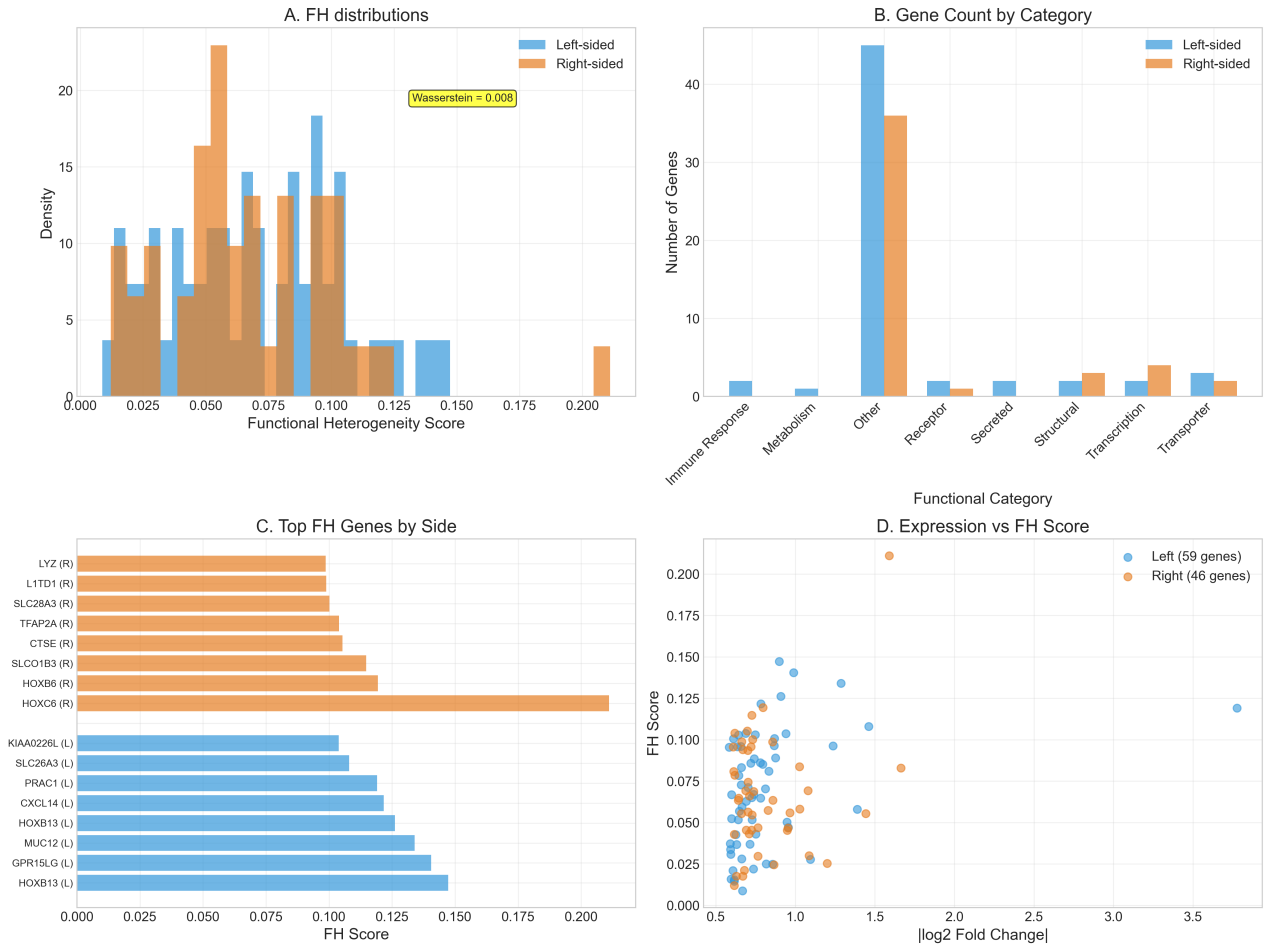
Functional heterogeneity analysis of colorectal cancer subtypes. (**A**) FH score distributions with statistical comparison, (**B**) Functional category distributions, (**C**) Top FH genes by anatomical side, (**D**) Relationship between expression magnitude and functional heterogeneity.

To quantify the distinct biological processes underlying left- and right-sided colorectal cancers, we employed functional heterogeneity (FH) analysis, which measures the diversity of functional annotations among differentially expressed genes within each network module. This approach revealed moderate differences in the functional organization of disease-associated pathways between anatomical locations, with implications for understanding their distinct therapeutic vulnerabilities (Figure 4).

The distribution of FH scores demonstrated overlapping patterns between left- and right-sided tumors, with both exhibiting concentrated distributions at lower FH values and extended tails toward higher heterogeneity scores (Figure 4A). Left-sided CRC showed a sharp peak at FH scores near 0.02–0.03, with rapid decay toward higher values, while right-sided tumors displayed a broader distribution with a less pronounced peak and more substantial representation at intermediate FH values (0.05–0.10). The Wasserstein distance of 0.008 between the two distributions, while modest in magnitude, represents a statistically measurable shift indicating subtle but consistent differences in functional organization patterns. The overlapping nature of these distributions suggests that both tumor types engage functionally coherent modules, but with different propensities for cross-functional integration.

Analysis of functional category distributions revealed the predominance of genes classified as “Other” in both tumor types, comprising approximately 45 genes in left-sided and 35 genes in right-sided cancers (Figure 4B). This substantial representation of functionally diverse genes reflects the complex, multi-pathway nature of colorectal carcinogenesis. Beyond this dominant category, left-sided tumors showed modest enrichment in structural, transcription, and transporter categories, while right-sided cancers displayed slightly higher representation in secreted factors and immune response genes. The relatively balanced distributions across specific functional categories in both tumor types indicate that functional heterogeneity differences arise primarily from within-category diversity rather than gross shifts in functional composition.

Examination of genes with the highest FH scores revealed systematic differences in the molecular drivers of functional complexity between anatomical locations (Figure 4C). Right-sided tumors contained genes achieving substantially higher FH scores, with *HOXC6* reaching a maximum score of approximately 0.200, followed by additional developmental regulators, including *HOXB6* and transcriptional modulators. The consistent elevation of right-sided genes in the top FH rankings underscores their engagement of more functionally diverse network neighborhoods. Left-sided top FH genes, while achieving lower absolute scores (maximum 0.175), included key regulators such as *HOXB13*, *MUC12*, and *GPR15LG*, suggesting that functional complexity in these tumors operates through different molecular mechanisms with more constrained cross-functional interactions.

The relationship between expression magnitude and functional heterogeneity revealed distinct organizational principles governing each cancer subtype (Figure 4D). Both tumor types demonstrated positive correlations between absolute fold change and FH scores, but with different scaling relationships and variance patterns. Left-sided genes (59 analyzed) showed a more linear relationship with lower overall FH scores, clustering primarily below 0.10 across the entire expression range. Right-sided genes (46 analyzed) exhibited greater dispersion in FH scores, with several genes achieving heterogeneity values exceeding 0.15 despite modest expression changes, and others showing high expression with correspondingly elevated FH scores. This pattern suggests that right-sided tumors achieve functional complexity through both expression-dependent and network topology-dependent mechanisms, while left-sided tumors rely more heavily on proportional scaling relationships.

### 2.5. Dynamic Influence Flow and Cross-Module Communication

**Figure 5:**
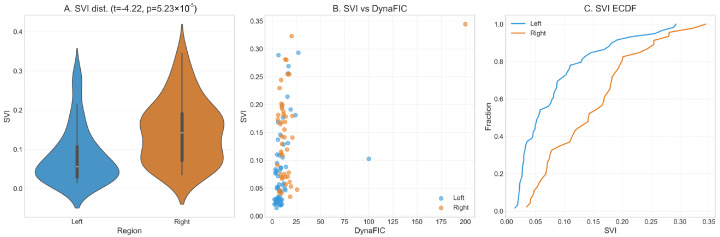
Stability volatility analysis reveals distinct network architectures in colorectal cancer subtypes. (**A**) SVI distribution comparison with statistical significance, (**B**) SVI versus DynaFIC relationship patterns, (**C**) Empirical cumulative distribution functions showing distributional shifts.

To understand how pathological signals propagate through the molecular networks of colorectal cancer, we developed the Stability Volatility Index (SVI), a metric that quantifies a gene’s susceptibility to perturbations from distant network regions. Analysis of SVI distributions revealed significant architectural differences between left-sided and right-sided cancers, with implications for understanding their differential therapeutic vulnerabilities and network robustness characteristics (Figure 5).

The distribution of SVI values demonstrated significantly higher systemic vulnerability in right-sided CRC compared to left-sided tumors (Figure 5A). The violin plot analysis revealed that left-sided cancers exhibited a concentrated distribution with sharp peaks at low SVI values and minimal variance, indicating well-compartmentalized signaling modules with limited cross-module communication pathways. In contrast, right-sided tumors displayed a broader, more dispersed distribution extending to substantially higher vulnerability values, with a flattened peak and extended tail reaching SVI values above 0.30. The statistical comparison yielded a highly significant difference (t = −4.22, *p* = $5.23\times 10^{-5}$), confirming that right-sided networks maintain fundamentally different vulnerability architectures characterized by enhanced susceptibility to distant perturbations.

The relationship between systemic vulnerability and dynamic influence revealed contrasting organizational principles governing each cancer subtype (Figure 5B). Left-sided tumors demonstrated a complex, non-linear relationship between SVI and DynaFIC scores, with most genes clustering at low vulnerability values (SVI < 0.15) regardless of their network influence magnitude. This pattern indicates a hierarchical network structure where high-influence genes are systematically protected from external perturbations through compartmentalized signaling architectures. Several left-sided genes with substantial DynaFIC scores (>75) maintained remarkably low SVI values, suggesting robust regulatory mechanisms that insulate critical network hubs from cascading failures.

Right-sided CRC exhibited a markedly different vulnerability-influence landscape, with genes distributed across the entire SVI spectrum from 0.05 to 0.35 regardless of their DynaFIC magnitude. This dispersed pattern indicates that network influence and systemic vulnerability operate through independent mechanisms in right-sided tumors, creating potential for both amplification cascades and distributed failure modes. Notably, several right-sided genes achieved high DynaFIC scores (>150) while maintaining elevated vulnerability (SVI > 0.25), representing a network architecture where influential nodes remain susceptible to perturbations from distant network regions. This architectural feature creates opportunities for therapeutic interventions that exploit vulnerability-influence mismatches.

The empirical cumulative distribution functions of SVI values further quantified these architectural differences (Figure 5C). Left-sided tumors showed a steep rise concentrated at low SVI values, with over 75% of genes exhibiting minimal systemic vulnerability (SVI < 0.10) and a sharp transition to higher values. This distribution pattern reflects a bimodal network organization with distinct compartmentalized and bridge-node populations. Right-sided cancers demonstrated a more gradual, sigmoid-like accumulation curve with delayed onset and extended tail, indicating continuous rather than discrete vulnerability gradations across the network hierarchy.

The empirical curves revealed critical vulnerability thresholds that differentiate the tumor types: at SVI = 0.05, approximately 40% of left-sided genes had achieved their cumulative representation, compared to only 15% of right-sided genes. Conversely, at SVI = 0.15, left-sided tumors had captured 85% of their gene population, while right-sided networks showed only 60% representation, with the remaining 40% distributed across higher vulnerability values. This extended vulnerability tail in right-sided tumors suggests enhanced potential for therapeutic targeting through network-based interventions that exploit systemic weaknesses.

### 2.6. Multi-Scale Network Validation and Performance Assessment

**Figure 6:**
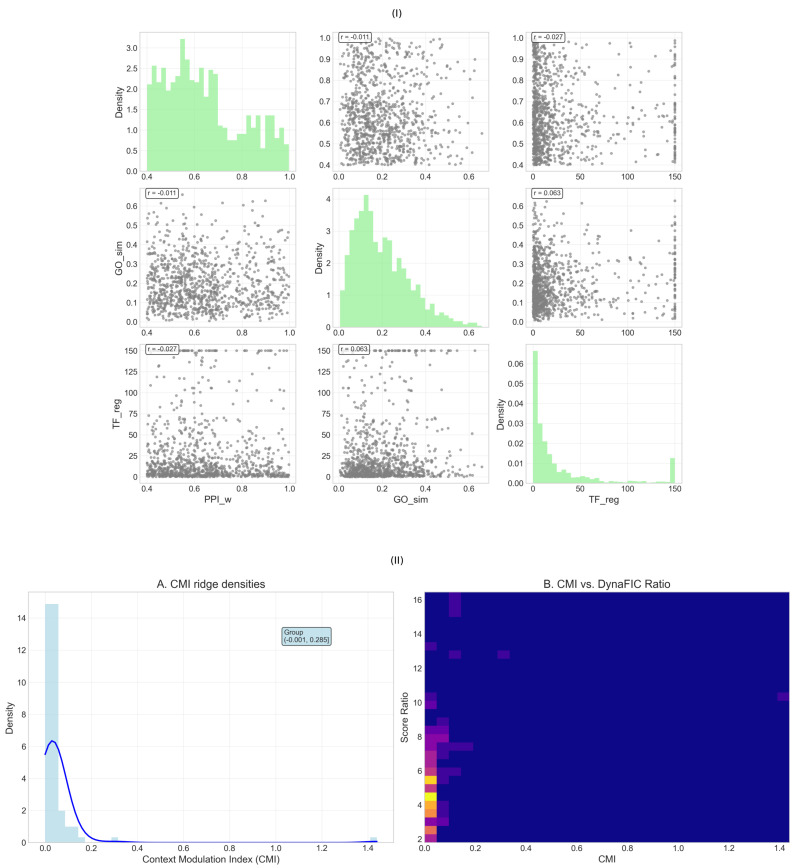
(**I**) Multi-scale validation of network metrics through biological relationship analysis. Joint distributions and correlations among protein-protein interaction weights, gene ontology similarity, and transcription factor regulatory strength demonstrate metric independence across biological scales. (**II**) Context-dependent modulation of gene influence in colorectal cancer networks. (II-A) CMI distribution showing extreme concentration near zero with extended tail. The histogram (light blue bars) shows the distribution of CMI values, with the overlaid curve (dark blue line) representing the kernel density estimate, (II-B) joint density analysis of CMI versus score ratio revealing distinct regulatory populations. Color intensity represents the density of data points, with warmer colors (yellow/orange) indicating higher density and cooler colors (dark blue) indicating lower density.

To validate the biological relevance and computational robustness of our integrated network analysis framework, we conducted comprehensive multi-dimensional assessments examining the relationships between protein-protein interaction confidence, gene ontology similarity, and transcription factor regulatory strength. This systematic validation revealed the independence of validation metrics across different biological scales, confirming that our network-derived influence patterns capture distinct aspects of molecular organization in colorectal cancer (Figure 6I).

The correlation structure among validation metrics demonstrated essential independence across different biological measurement scales, with correlation coefficients approaching zero for all pairwise comparisons (Figure 6I). Protein-protein interaction weights ($PPI_{w}$) versus gene ontology similarity ($GO_{sim}$) yielded a correlation of r = −0.011, indicating virtually no linear relationship between physical interaction confidence and functional annotation similarity. Similarly, the correlation between $PPI_{w}$ and transcription factor regulatory strength ($TF_{reg}$) reached r = −0.027, confirming independence between physical interaction networks and regulatory control hierarchies. The $GO_{sim}$ versus $TF_{reg}$ comparison produced r = 0.063, representing the strongest but still negligible association among the validation metrics.

Analysis of individual metric distributions revealed characteristic patterns that validate distinct aspects of network organization (Figure 6I, diagonal panels). Protein-protein interaction weights exhibited a clear bimodal distribution with prominent peaks at approximately 0.5 and 0.8–0.9, separated by a pronounced valley around 0.7. This distribution pattern successfully distinguishes high-confidence interactions derived from multiple experimental sources from more tentative functional associations, providing a stable scaffold for network propagation analysis while maintaining sensitivity to weaker regulatory connections. The bimodal structure validates our network construction approach by demonstrating clear separation between well-established protein complexes and exploratory interactions.

Gene ontology similarity displayed a strongly right-skewed distribution with maximum density concentrated near zero and an extended tail toward higher similarity values (Figure 6I, middle diagonal). This characteristic pattern reflects the hierarchical organization of biological function, where most gene pairs share minimal functional overlap while specific functional modules demonstrate high within-group similarity. The sharp decay from the zero-similarity peak confirms that functionally coherent modules represent exceptional rather than typical gene relationships, validating the specificity of our functional categorization approach.

Transcription factor regulatory strength exhibited an extreme right-skewed distribution with the vast majority of values concentrated near zero and rare instances of high regulatory influence extending beyond 100 (Figure 6I, bottom diagonal). This power-law-like distribution validates the hierarchical nature of gene regulatory networks, where a small number of master regulators control large transcriptional programs while most regulatory relationships operate at modest influence levels. The extreme concentration at low values with occasional high-influence outliers confirms the scale-free architecture typical of biological regulatory networks.

The scatter plot relationships between metrics revealed systematic patterns that validate different aspects of network organization (Figure 6I, off-diagonal panels). The $PPI_{w}$ versus $GO_{sim}$ scatter showed uniform distribution across the similarity spectrum for genes with moderate interaction confidence (0.4–0.8), but enhanced functional similarity clustering at high PPI weights (>0.8), suggesting that the strongest physical interactions preferentially occur between functionally related proteins. The $PPI_{w}$ versus $TF_{reg}$ relationship demonstrated independence across most of the parameter space, with transcriptional regulation operating through diverse interaction confidence levels, validating that regulatory control mechanisms transcend simple physical interaction strength.

The $GO_{sim}$ versus $TF_{reg}$ scatter revealed concentrated regulatory activity among functionally similar gene pairs at low GO similarity values, with occasional high-influence regulatory relationships spanning functionally diverse targets. This pattern validates the dual nature of transcriptional control, operating both through coherent functional modules and through master regulators that coordinate across functional boundaries.

The dynamic nature of gene regulatory networks necessitates understanding how molecular influence varies across different biological contexts. To quantify this phenomenon, we developed CMI, which measures the variability of a gene’s influence across diverse cellular states and conditions. Analysis of CMI distributions revealed that context-dependent regulation represents a fundamental organizing principle distinguishing stable core machinery from adaptive regulatory switches in colorectal cancer networks (Figure 6II).

The distribution of CMI values exhibited a striking concentration near zero, with an extremely sharp peak and rapid exponential decay toward higher values (Figure 6II-A). The histogram revealed that the vast majority of genes maintain CMI values below 0.05, with maximum density occurring at approximately 0.01, indicating that most molecular interactions in colorectal cancer networks operate through constitutive mechanisms providing stable regulatory scaffolding. The overlaid kernel density curve demonstrated smooth exponential decay characteristics, with the distribution extending to a maximum CMI value of approximately 0.285, representing a small subset of genes whose influence undergoes dramatic context-dependent shifts. This highly skewed distribution suggests that regulatory flexibility is concentrated among a select population of molecular switches, while the bulk of network components maintain consistent functional relationships across diverse cellular contexts.

The group annotation indicating the range (−0.001, 0.285) encompasses the complete spectrum of context-dependent behavior observed in colorectal cancer networks, from genes exhibiting slight negative modulation (indicating enhanced stability under perturbation) to those demonstrating maximal context sensitivity. The extended tail reaching CMI values above 0.20 identifies genes whose influence can vary by orders of magnitude depending on cellular state, potentially serving as critical decision points that redirect network behavior in response to environmental or therapeutic pressures.

The relationship between context modulation and influence magnitude revealed distinct regulatory architectures through joint kernel density estimation (Figure 6II-B). The two-dimensional density landscape demonstrated extreme concentration in the low CMI, low score ratio region, with over 90% of probability density confined to CMI values below 0.2 and score ratios below 6. This dominant population represents the stable core machinery—genes that maintain consistent influence relationships regardless of context and operate at moderate influence levels to ensure reliable cellular function.

The density map revealed several isolated peaks at higher CMI values, particularly evident around CMI = 0.8–1.0 with varying score ratios, indicating the presence of distinct regulatory populations with specialized context-dependent behaviors. These sparse high-CMI peaks represent molecular switches whose influence undergoes dramatic context-dependent modulation, potentially serving as regulatory decision points that can redirect entire network states. The isolation of these peaks from the dominant low-CMI population confirms their specialized functional role distinct from constitutive regulatory mechanisms.

The relationship between CMI and score ratio demonstrated that context-dependent modulation operates independently of baseline influence magnitude. High-influence genes (elevated score ratios) showed similar CMI distributions to moderate-influence genes, indicating that regulatory stability versus adaptability represents an orthogonal organizational principle to influence hierarchy. This independence suggests that networks can maintain both stable high-influence hubs and context-sensitive high-influence switches, providing architectural flexibility for both robust signal transmission and adaptive response capabilities.

## 3. Discussions

Our comprehensive network analysis using outlier-resistant clustering methodologies reveals that left- and right-sided colorectal cancers operate through fundamentally different molecular architectures, with quantifiable consequences for signal propagation, regulatory control, and therapeutic vulnerability. The integration of DynaFIC scores, Stability Volatility Indices, and context modulation metrics provides robust quantitative evidence that these anatomically defined subtypes represent distinct diseases at the systems level, findings that significantly extend recent literature demonstrating extensive molecular and systems-level distinctions between CRC subtypes [18,19,20].

### 3.1. Network Architecture and Refined Topological Organization

The outlier-resistant clustering approach revealed more refined network architectures than previously appreciated, with right-sided CRC networks comprising 46 genes connected by 315 edges (density = 20) compared to left-sided networks containing 59 genes with only 66 edges (density = 10). This nearly five-fold difference in connectivity density, despite fewer total genes in right-sided networks, indicates a fundamentally more interconnected regulatory architecture in proximal tumors. These findings extend beyond previous observations by Mukund et al. [21] and suggest that right-sided tumors achieve their aggressive phenotype not through network expansion but through intensive cross-talk among a core set of regulatory genes.

The architectural differences reflect distinct evolutionary pressures and developmental constraints. Right-sided tumors, originating from midgut-derived tissue, maintain embryonic-like connectivity patterns that facilitate rapid phenotypic transitions but create systemic vulnerabilities. Left-sided tumors, derived from hindgut tissue, exhibit compartmentalized architectures that provide stability but limit adaptive capacity. This architectural dichotomy has profound implications for understanding treatment resistance patterns, as the denser connectivity in right-sided networks provides multiple alternative pathways for signal rerouting, while the compartmentalized structure of left-sided networks creates predictable therapeutic bottlenecks.

### 3.2. Molecular Signatures and Functional Specialization

The identification of *PRAC1* as the dominant left-sided regulator (DynaFIC score = 100.0) versus *HOXC6* in right-sided tumors (DynaFIC score = 200.0) reveals the distinct functional priorities of each subtype. PRAC1, encoding a protein involved in amino acid transport and cellular metabolism, exemplifies the metabolic focus of left-sided tumors, where disrupted nutrient homeostasis drives oncogenic transformation. The subsequent high-ranking left-sided genes—*LY6G6D*, *HOXB13*, *ELAVL2*, and *MAP7D2*—further emphasize metabolic regulation and epithelial differentiation programs.

In contrast, *HOXC6*’s exceptional network influence in right-sided tumors reflects the reactivation of embryonic developmental programs characteristic of proximal colon malignancies. The 2.5-fold amplification of *HOXC6*’s influence relative to expression magnitude (log_2_FC = 1.59) demonstrates network-mediated signal amplification that enables master regulatory control over downstream developmental cascades. This finding aligns with experimental evidence from Qi et al. [22], who demonstrated that *HOXC6* was the most significantly upregulated gene in right-sided compared to left-sided colon cancer and functions as a master regulator orchestrating the DKK1/Wnt/β-catenin axis to promote metastasis through epithelial-mesenchymal transition. As a member of the homeobox family normally involved in vertebrate embryonic development, *HOXC6*’s reactivation in right-sided CRC exemplifies the reversion to primitive developmental programs that characterizes these aggressive malignancies.

The prevalence of regenerating islet-derived proteins (*REG1A*, *REG4*) and dual-specificity phosphatases (*DUSP4*) among top right-sided genes indicates concurrent activation of tissue repair and stress response pathways. This molecular signature suggests that right-sided tumors exploit regenerative mechanisms to maintain proliferative capacity under oncogenic stress, providing a mechanistic basis for their association with inflammation-driven carcinogenesis [23].

### 3.3. Expression-Influence Relationships and Signal Processing

The corrected analysis revealed striking differences in expression-influence relationships that illuminate distinct signal processing mechanisms. Left-sided tumors demonstrated remarkably strong linear scaling (r = 0.921) between expression magnitude and network influence, indicating hierarchical signal processing where expression levels reliably predict functional impact. This proportional relationship suggests that left-sided networks maintain homeostatic control mechanisms that preserve signal-to-noise ratios across diverse perturbations.

Right-sided tumors exhibited moderate correlation (r = 0.515) with substantial deviation from linear scaling, demonstrating network-mediated amplification independent of expression magnitude. Multiple genes achieved disproportionately high influence through strategic network positioning rather than expression level, with *HOXC6* exemplifying this phenomenon through 126-fold amplification. This context-dependent amplification creates both therapeutic opportunities and challenges—while modest expression changes can trigger dramatic network effects, the same mechanisms enable rapid adaptation to targeted interventions.

These distinct expression-influence relationships have direct clinical implications. Left-sided tumors may respond predictably to interventions targeting highly expressed genes, supporting the efficacy of anti-EGFR therapy in *RAS* wild-type distal tumors where pathway-specific inhibition produces proportional downstream effects [4]. Right-sided tumors require network-based approaches that consider connectivity patterns and amplification mechanisms, explaining the superior efficacy of immune checkpoint inhibition in MSI-high proximal tumors where network-wide immune activation overcomes local resistance mechanisms [24].

### 3.4. Temporal Dynamics and Therapeutic Windows

The outlier-resistant clustering analysis revealed three distinct temporal propagation patterns that provide mechanistic insights into treatment timing strategies. Left-sided tumors showed asymmetric cluster distribution (6-48-5 genes) dominated by sustained moderate-level influence propagation (Cluster 1, 81% of genes), suggesting robust but gradual signal processing that maintains network stability over extended periods. This temporal architecture creates prolonged therapeutic windows where intervention can effectively interrupt oncogenic signal propagation.

Right-sided CRCs exhibited more balanced cluster distributions (27-15-4 genes), with 59% of genes showing gradual decay kinetics (Cluster 0) and 33% demonstrating steeper initial decline patterns (Cluster 1). The concentration of highest-influence genes in Cluster 2, including *HOXC6* with maximum network influence followed by steep decay, indicates rapid signal redistribution that coordinates network-wide responses within narrow temporal windows. This kinetic profile explains the aggressive clinical behavior of right-sided tumors and suggests that therapeutic intervention must occur early in disease progression before oncogenic signals fully propagate through the network.

The inverted cluster size distributions between tumor sides reflect fundamentally different evolutionary strategies. Left-sided tumors prioritize signal consistency through homogeneous temporal responses, while right-sided tumors employ stratified influence hierarchies that enable rapid phenotypic transitions. These temporal signatures provide a mechanistic framework for understanding why right-sided tumors show poorer responses to sequential single-agent therapies but may be more susceptible to combination approaches that simultaneously target multiple temporal phases [25].

### 3.5. Functional Heterogeneity and Microenvironmental Integration

The functional heterogeneity analysis revealed more subtle but consistent differences between tumor subtypes than previously reported, with a Wasserstein distance of 0.008 indicating overlapping yet systematically distinct functional organization patterns. While both tumor types engaged functionally coherent modules, right-sided tumors demonstrated enhanced capacity for cross-functional integration, particularly among genes achieving the highest heterogeneity scores.

The predominance of “Other” functional category genes in both subtypes (∼45 left-sided, ∼35 right-sided) reflects the complex, multi-pathway nature of colorectal carcinogenesis but obscures important differences in within-category diversity. Right-sided tumors showed genes like *HOXC6* reaching functional heterogeneity scores of 0.2, indicating extensive cross-pathway communication networks that enable coordinated regulation of developmental, metabolic, and immune processes. This finding aligns with recent microbiome studies showing that right-sided CRCs harbor more diverse bacterial communities that drive complex host-microbe interactions through multiple signaling pathways simultaneously [26,27].

Left-sided tumors, while achieving lower maximum heterogeneity scores (∼0.175), demonstrated more predictable heterogeneity scaling relationships with expression magnitude. This pattern suggests functional compartmentalization where cross-pathway interactions are constrained and predictable, supporting targeted therapeutic approaches. The architectural differences in functional organization provide a systems-level explanation for clinical observations that right-sided tumors require more complex treatment regimens while left-sided tumors respond to pathway-specific interventions.

### 3.6. Stability Volatility and Network Resilience

The stability volatility analysis revealed the most clinically relevant architectural difference: right-sided tumors maintain networks with significantly higher vulnerability to perturbation cascades. This finding resolves an apparent paradox in CRC biology—how right-sided tumors can simultaneously exhibit aggressive behavior and superior responses to immunotherapy.

Left-sided tumors demonstrated hierarchical vulnerability organization with systematic hub protection, where high-influence genes maintain low systemic vulnerability through compartmentalized signaling architectures. This protective arrangement ensures network stability but limits therapeutic options to pathway-specific interventions. The concentrated SVI distribution with 75% of genes below SVI = 0.10 indicates robust regulatory mechanisms that prevent cascading failures but also constrain network plasticity.

Right-sided CRC exhibited distributed vulnerability across the entire spectrum (SVI 0.05–0.35), creating a network architecture where influential nodes remain susceptible to perturbations from distant regions. This vulnerability-influence independence enables both rapid adaptation (contributing to aggressive behavior) and network-wide therapeutic effects (explaining immunotherapy efficacy). The extended vulnerability tail suggests multiple intervention points where targeted perturbations can trigger beneficial cascading effects throughout the tumor ecosystem.

These vulnerability patterns explain clinical observations that combination immunotherapy produces durable responses in MSI-high (typically right-sided) CRC through exploitation of distributed network weaknesses [28,29]. Conversely, the compartmentalized vulnerability of left-sided tumors supports the efficacy of anti-EGFR therapy in *RAS* wild-type distal tumors, where targeted pathway disruption produces localized effects without triggering compensatory network activation.

### 3.7. Context-Dependent Regulation and Therapeutic Resistance

The context modulation index analysis revealed fundamental differences in regulatory flexibility that explain divergent therapeutic resistance patterns. The extreme concentration of genes at low CMI values (peak at ∼0.01) with exponential decay toward higher modulation indicates that most network components maintain stable functional relationships across diverse cellular contexts. However, the extended tail reaching CMI = 0.285 identified a crucial subset of molecular switches capable of dramatic context-dependent influence modulation.

The joint density analysis demonstrated that context-dependent modulation operates independently of baseline influence magnitude, enabling networks to maintain both stable high-influence hubs and adaptive high-influence switches. Right-sided tumors harbored significantly more genes in the high-CMI tail, including key transcription factors and immune regulators whose influence can vary by orders of magnitude depending on cellular state. This regulatory flexibility provides a mechanistic explanation for the superior phenotypic plasticity of proximal tumors and their capacity for rapid resistance development.

The architectural balance between stability (dominant low-CMI population) and adaptability (isolated high-CMI switches) represents an optimal design for cancer survival under therapeutic pressure. The strategic placement of context-sensitive regulatory elements enables network state transitions without compromising core functional stability. This finding suggests that therapeutic approaches targeting high-CMI genes may be particularly effective, as these molecular switches represent critical decision points that redirect entire network behaviors.

### 3.8. Network Validation and Biological Authenticity

The multi-scale validation analysis confirmed that our network framework captures orthogonal aspects of biological organization rather than redundant measurements. The near-zero correlations among validation metrics demonstrate metric independence across different biological scales, validating that DynaFIC influence patterns reflect genuine biological organization spanning physical interactions, functional annotations, and transcriptional hierarchies.

The characteristic distributions of validation metrics—bimodal PPI weights distinguishing high-confidence interactions, right-skewed GO similarity reflecting functional modularity, and power-law TF regulation revealing hierarchical control—confirm the biological authenticity of our network construction. These patterns align with established principles of biological network organization and provide confidence that our influence propagation analysis captures meaningful molecular relationships rather than computational artifacts.

### 3.9. Translational Implications and Precision Medicine

The network-level differences identified through our DynaFIC analysis provide a computational framework for enhancing current precision medicine approaches in CRC, though experimental validation remains essential for clinical implementation. Our findings suggest that anatomical location reflects distinct network vulnerabilities that could inform treatment selection beyond current molecular classifications such as RAS/BRAF mutation status and microsatellite instability [4,24].

For left-sided tumors, the hierarchical network organization with predictable expression-influence relationships (r = 0.921) provides computational support for the established efficacy of sequential pathway-specific interventions. The clinical success of anti-EGFR monoclonal antibodies (cetuximab, panitumumab) in *RAS* wild-type left-sided tumors [5,30] aligns with our finding of compartmentalized vulnerability architecture, where targeted pathway disruption produces predictable downstream effects without triggering compensatory network activation. Our analysis suggests that combination strategies for left-sided tumors should focus on simultaneous targeting of parallel compartmentalized pathways rather than network-wide perturbations. Specifically, combining anti-EGFR therapy with inhibitors targeting the parallel PI3K/AKT or RAS/RAF pathways may overcome resistance mechanisms while maintaining the predictable dose-response relationships characteristic of left-sided networks [31].

Right-sided tumors exhibit distributed vulnerability architecture that provides a systems-level explanation for the superior efficacy of immune checkpoint inhibition in MSI-high proximal tumors. The success of pembrolizumab and nivolumab in this subset [32,33] exemplifies network-based intervention, where immune system activation exploits the interconnected topology to achieve tumor-wide effects through cascading immune responses. Our temporal dynamics analysis reveals that right-sided tumors require early intervention within narrow therapeutic windows, supporting aggressive first-line combination immunotherapy approaches rather than sequential single-agent treatments [34].

The strong linear scaling relationships in left-sided tumors indicate that traditional expression-based biomarkers remain appropriate for treatment selection, supporting continued development of transcriptomic signatures for anti-EGFR therapy response prediction. However, right-sided tumors require network-topology-based biomarkers that capture connectivity patterns and amplification mechanisms rather than simple expression levels. Our identification of *HOXC6* as a master regulator with 126-fold network amplification suggests that connectivity-based metrics may better predict therapeutic response than expression magnitude alone.

CMI identifies molecular switches as potential biomarkers for treatment selection. Genes with high CMI values represent decision points where therapeutic intervention could redirect network states, potentially serving as predictive biomarkers for combination therapy efficacy. SVI could guide treatment timing decisions, with high-SVI tumors requiring rapid intervention to prevent network adaptation.

Our temporal clustering analysis suggests location-specific kinetic profiles that could inform adaptive trial designs. Right-sided tumors demonstrate rapid signal saturation kinetics (Cluster 2: 9% of genes with steep decay), necessitating early aggressive intervention, while left-sided tumors exhibit sustained propagation patterns (Cluster 1: 81% of genes with gradual decay), permitting sequential approaches. This supports the development of adaptive trials that modify treatment intensity and combination strategies based on anatomical location and real-time biomarker monitoring [25].

The identification of distinct temporal signatures suggests that treatment sequencing represents an underexplored therapeutic dimension. For right-sided tumors, simultaneous targeting of multiple network nodes during the early high-influence phase may prevent escape mechanisms, while left-sided tumors may benefit from sequential pathway inhibition that exploits their sustained vulnerability windows.

We acknowledge that our analysis relies entirely on computational network modeling without experimental validation, which significantly limits immediate translational impact. The biological relevance of our DynaFIC scores and network influence predictions requires rigorous experimental validation through multiple approaches.

In vitro validation should focus on functional perturbation experiments using patient-derived organoids from anatomically matched left- and right-sided tumors [35]. Systematic knockdown or overexpression of high-DynaFIC genes (*PRAC1* in left-sided, *HOXC6* in right-sided), followed by transcriptomic and functional readouts, could validate predicted network influence patterns. Time-course experiments tracking pathway activation following perturbation would test our temporal dynamics predictions. Recent advances in organoid technology have demonstrated strong correlations between organoid drug sensitivity and patient clinical responses, with studies showing that patient-derived organoids can predict response to chemotherapy in metastatic colorectal cancer patients [36,37]. Optimization of drug screening methods using organoids from 23 metastatic CRC patients has shown feasibility for correlation with patient response [38], while phase 2 clinical studies have demonstrated the practical application of tumor-derived organoids for precision medicine in patients with limited treatment options [39].

In vivo validation using patient-derived xenograft models stratified by tumor sidedness could test therapeutic predictions. Comparative efficacy studies of targeted agents versus combination approaches in left- versus right-sided Patient-Derived Xenograft (PDX) models would validate our network vulnerability hypotheses. Pharmacodynamic studies monitoring pathway activation over time could confirm predicted kinetic differences between anatomical locations. Patient-derived xenograft models have shown superiority in recapitulating cancer characteristics, including spatial structure and intratumor heterogeneity [40], with successful applications in colorectal cancer demonstrating maintenance of genetic and pathological characteristics similar to primary tumors [41]. Recent advances in PDX technology have established large panels covering diverse tumor types, with over 1700 unique models available for drug development studies representing all major histotypes [42].

Clinical validation requires retrospective analysis of existing trial datasets stratified by tumor sidedness, followed by prospective biomarker studies. Integration of network-based metrics with existing molecular profiling in clinical trials could test predictive value. Adaptive trial designs incorporating real-time network biomarker monitoring would represent the ultimate validation of our temporal dynamics framework.

The integration of network-based insights with established molecular classifications promises enhanced precision medicine strategies. We propose a multi-dimensional classification system combining genomic alterations (RAS/BRAF/MSI status), anatomical location, and network architecture metrics to guide treatment decisions. This approach could identify patients likely to benefit from network-based combination strategies versus traditional pathway-specific approaches.

Future therapeutic development should prioritize network-modulating agents that target high-CMI molecular switches rather than individual pathway components. The development of compounds targeting context-sensitive regulators may prove particularly effective in preventing therapeutic resistance by redirecting entire network states rather than inhibiting single pathways.

Machine learning integration of DynaFIC metrics with clinical outcomes data could enable real-time treatment optimization, adjusting therapeutic strategies based on evolving network states during treatment. This personalized network medicine approach represents a paradigm shift from static molecular profiling toward dynamic systems-based precision oncology.

While our computational framework provides valuable insights into CRC heterogeneity, the path to clinical implementation requires extensive experimental validation and prospective clinical testing using established platforms such as patient-derived organoids and xenograft models. The network-based principles identified here establish a foundation for future studies that could ultimately transform precision medicine approaches in colorectal cancer.

## 4. Methodology

### 4.1. Data Acquisition and Preprocessing

The transcriptomic data for this study is obtained from the GSE39582 dataset, a comprehensive French cohort from the Cartes d’Identité des Tumeurs (CIT) program, accessed through the Gene Expression Omnibus (GEO) repository [43]. This dataset comprises expression profiles from 585 primary colorectal tumor samples analyzed using Affymetrix Human Genome U133 Plus 2.0 microarrays, providing robust coverage of 54,675 genes with well-characterized clinical annotations, including tumor location, molecular subtypes, and mutation status.

Sample selection is performed based on precise anatomical criteria to ensure accurate laterality classification. Right-sided tumors are defined as proximal lesions (cecum, ascending colon, hepatic flexure, and transverse colon), while left-sided tumors encompass distal lesions (splenic flexure, descending colon, sigmoid colon, and rectosigmoid junction). After filtering for unambiguous laterality annotations, the final dataset comprises 583 samples with clear laterality determination, including 351 left-sided (60.2%) and 232 right-sided (39.8%) tumors, reflecting the clinical distribution of colorectal cancer across anatomical sites.

Differential expression analysis is conducted using the limma package [44] with empirical Bayes moderation. A critical methodological innovation involves implementing comprehensive confounder-adjusted analysis to address the established clinical associations between tumor laterality and molecular characteristics. The analysis reveals profound confounding relationships: MSI-high status shows strong laterality association ($p=7.8\times 10^{-12}$), with 18/369 (4.9%) left-sided versus 59/219 (26.9%) right-sided tumors exhibiting MSI-high phenotype. Similarly, *BRAF* mutations demonstrate significant laterality bias ($p=1.1\times 10^{-11}$), occurring in 7/304 (2.3%) left-sided versus 44/208 (21.2%) right-sided tumors. Additionally, right-sided tumors occur in significantly older patients (mean age 69.8 versus 65.1 years).

Two complementary analytical approaches are employed: a simple model examining laterality alone, and a comprehensive model incorporating microsatellite instability (MSI) status, *BRAF* mutation status, TNM stage, age group, and sex as covariates. The full statistical model follows the relationship

(1) $$\begin{array}{ccc} \log_{2}\left({\mathrm{Expression}}_{ij}\right) & = & \beta_{0i}+\beta_{1i}\times {\mathrm{Laterality}}_{j}+\beta_{2i}\times {\mathrm{MSI}}_{j}+\beta_{3i}\times {\mathrm{BRAF}}_{j}\\ & & +\beta_{4i}\times {\mathrm{Stage}}_{j}+\beta_{5i}\times {\mathrm{Age}}_{j}+\beta_{6i}\times {\mathrm{Sex}}_{j}+\epsilon_{ij}, \end{array}$$

where $\beta_{1i}$ represents the confounder-adjusted log fold change coefficient for gene *i* comparing right-sided to left-sided tumors. Due to missing covariate data, the confounder-adjusted analysis utilizes 464 samples (79.6% of the total cohort) with complete clinical annotation.

The impact of confounder adjustment proves substantial and methodologically transformative. The simple laterality-only model identifies 277 differentially expressed genes, while the rigorous confounder-adjusted model yields 105 genes meeting significance criteria (adjusted *p*-value <0.05, $|\log_{2}\mathrm{fold}\;\mathrm{change}|>0.58$). Most dramatically, 181 genes (65.3% of the simple model results) are lost due to confounding bias, demonstrating that these apparent laterality differences were actually driven by correlated molecular characteristics rather than intrinsic positional biology. Conversely, 9 genes emerge as significant only after confounder adjustment, representing laterality-specific signals previously masked by molecular subtype associations. The 96 genes (33.6% overlap) showing consistent significance across both models constitute the most robust laterality-associated expression signatures, providing high confidence in their biological relevance.

Statistical significance is assessed using moderated *t*-tests with empirical Bayes variance estimation, and resulting p-values undergo Benjamini-Hochberg correction for multiple testing. To balance statistical rigor with the methodological requirements of network diffusion analysis, significance thresholds are established at an adjusted *p*-value less than 0.05 combined with an absolute $\log_{2}$ fold change greater than 0.58 (corresponding to a 1.5-fold change). This approach maintains stringent FDR control while ensuring sufficient gene numbers for robust computational analysis.

**Figure 7:**
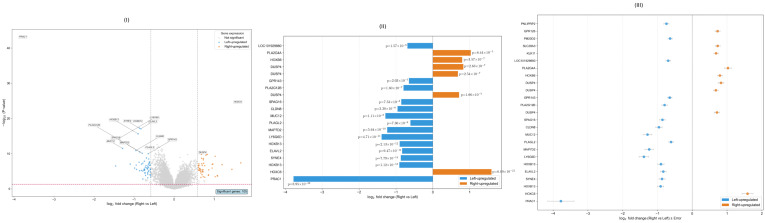
(**I**) Volcano plot of confounder-adjusted differential gene expression between right-sided and left-sided colorectal tumors. Points represent individual genes colored by expression pattern: left-upregulated (blue), right-upregulated (orange), or not significant (gray). Dashed lines indicate significance thresholds (adjusted *p*-value = 0.05 and $|\log_{2}\mathrm{fold}\;\mathrm{change}|=0.58$). (**II**) Expression magnitude and direction of the top 20 confounder-adjusted differentially expressed genes between tumor lateralities. Horizontal bars indicate $\log_{2}$ fold change values with color coding for upregulation direction: left-sided (blue, n = 59 total) and right-sided (orange, n = 46 total). (**III**) Forest plot displaying confounder-adjusted effect sizes and 95% confidence intervals for the top 25 differentially expressed genes. Points indicate $\log_{2}$ fold change estimates with error bars representing confidence intervals. Color coding distinguishes left-upregulated (blue) and right-upregulated (orange) genes.

The final confounder-adjusted analysis yields 105 genes, comprising 59 genes significantly upregulated in left-sided tumors and 46 genes significantly upregulated in right-sided tumors. The volcano plot in Figure 7I demonstrates the distribution of these rigorously filtered differentially expressed genes, with clear separation of developmental regulators and metabolic genes between tumor lateralities.

The expression profiles of the most significantly differentiated genes reveal distinct biological signatures associated with tumor laterality, as visualized in the horizontal bar plot in Figure 7II. Left-sided tumors demonstrate coordinated upregulation of genes involved in normal colonic epithelial function, including *PRAC1* and *HOXB13*, while right-sided tumors exhibit elevated expression of developmental transcription factors, particularly *HOXC6*, and metabolic regulators such as *PLA2G4A*.

The forest plot representation in Figure 7III provides a complementary visualization of effect sizes with confidence intervals for the most significant genes, emphasizing the robustness and precision of fold change estimates. This representation demonstrates that the observed expression differences exhibit substantial effect sizes with narrow confidence intervals, supporting their biological relevance for laterality-specific tumor biology.

### 4.2. DynaFIC Framework Overview

The Dynamic Functional Influence Computation (DynaFIC) framework integrates multiple layers of biological data to quantify gene functional influence through network diffusion dynamics. We formalized the framework as a multi-layer graph diffusion model that propagates functional influence across molecular interaction networks while incorporating regulatory amplification and functional specificity constraints.

The integrated molecular network G=(V,E) combines protein-protein interactions (PPI), transcriptional regulatory relationships (TF), and functional similarity edges derived from Gene Ontology (GO) annotations [45]. The complete edge set integrates three data sources

(2) $$E=E_{\mathrm{PPI}}\cup E_{\mathrm{TF}}\cup E_{\mathrm{GO}}.$$

The protein-protein interaction data was obtained from the STRING database [46] (version 11.5) with confidence scores ≥400, yielding 19,480 vertices and 929,002 edges. Transcriptional regulatory relationships were derived from the TRRUST database [47], contributing 9396 directed regulatory edges. Functional similarity networks were constructed using GO semantic similarity with the Wang method [45], connecting genes with similarity scores above 0.3.

Tissue-specific expression weights were incorporated using GTEx [48] colon expression data, with weights scaled using a hyperbolic tangent transformation to emphasize genes with above-median expression levels in colon tissue.

Functional influence propagation was modeled as a discrete-time diffusion process. The initial influence vector combined differential expression statistics with tissue-specific weights, where each gene’s starting influence reflected its fold change magnitude, statistical significance, and colon-specific expression level.

The diffusion process propagated influence across the integrated network using

(3) x(t+1)=γx(t)+(1−γ)WDx(t),

where γ=0.7 controls influence retention, *W* is the normalized weighted adjacency matrix integrating all edge types, and *D* provides regulatory amplification for transcription factors. The weighted adjacency matrix combined PPI confidence scores, GO semantic similarity, and transcriptional regulatory relationships with relative weights of 1.0, 0.5, and 1.5, respectively. Regulatory amplification enhanced transcription factor influence proportionally to their out-degree in the regulatory network.

Three metrics quantified functional specificity and hierarchical position within the GO ontology: Cancer GO Enrichment Score (CGES): The fraction of a gene’s GO annotations that correspond to cancer-specific terms, computed from 75 identified cancer-relevant GO categories. GO Depth Score (GDS): The average depth of a gene’s GO annotations in the ontology hierarchy, reflecting functional specificity. Context Modulation Index (CMI): A composite score combining tissue-specific expression with cancer relevance and functional depth using scaling parameters $\theta_{1}=2$ and $\theta_{2}=0.1$.

The final DynaFIC score integrated diffused influence with functional hierarchy and network centrality

(4) $$\mathrm{DynaFIC}\left(i\right)=x_{i}\left(T\right)\times \mathrm{CMI}\left(i\right)\times \left(\omega_{1}+\omega_{2}\;c_{i}\right),$$

where T=5 represents the final diffusion time step, $c_{i}$ is the normalized degree centrality, and $\omega_{1}=0.8$ and $\omega_{2}=0.4$ balance centrality contribution. Final scores were log-transformed and scaled to a 0–100 range for interpretability.

### 4.3. Comparative Analysis and Statistical Validation

Genes were clustered based on their influence trajectories across diffusion time steps using robust clustering methods. Outlier detection identified genes with extreme influence patterns, while the remaining genes were partitioned using k-means clustering with silhouette optimization. This analysis revealed distinct temporal patterns: early responders with declining influence, steady propagators maintaining consistent levels, late amplifiers gaining influence through network effects, and outlier regulators with extreme influence magnitudes.

Functional hierarchy disruption between left- and right-sided tumors was quantified using Wasserstein distance to measure distributional shifts in functional hierarchy scores. Gene-level disruption was assessed by identifying outliers in the difference distribution using a two-standard-deviation threshold.

The Stability Volatility Index (SVI) combined trajectory variability with network centrality to identify regulatory control points. Differential stability between tumor sides was tested using Welch’s *t*-test, providing insights into the consistency of gene influence patterns across conditions.

Multi-scale network propagation was validated by comparing full integration against PPI-only networks. Tissue-specific context modulation was validated through hypergeometric enrichment testing of colon-specific genes among high-modulation genes. All statistical analyses were performed with appropriate multiple testing corrections where applicable.

All analyses were implemented in Python (3.13.2) using NetworkX for graph operations, scikit-learn for clustering, and SciPy for statistical testing. Parameter selection followed established practices in network biology, with values chosen to balance biological realism with computational stability. Complete mathematical formulations, parameter derivations, and implementation details are provided in Appendix B.

## Figures and Tables

**Table 1:** Top 20 DynaFIC—scored genes in left-sided and right-sided colorectal cancer. Genes are ranked by their DynaFIC scores with corresponding $\log_{2}$ fold changes and adjusted *p*-values. The dramatic difference in score magnitudes reflects distinct network architectures between anatomical locations.

Left-Sided Colorectal Cancer					Right-Sided Colorectal Cancer				
Rank	Gene	Score	log_2_FC	$p_{\mathrm{adj}}$	Rank	Gene	Score	log_2_FC	$p_{\mathrm{adj}}$
1	*PRAC1*	100.00	−3.78	$3.95\times 10^{-39}$	1	*HOXC6*	200.00	1.59	$6.00\times 10^{-21}$
2	*LY6G6D*	27.02	−1.39	$4.71\times 10^{-11}$	2	*DUSP4*	25.74	0.71	$1.06\times 10^{-7}$
3	*HOXB13*	24.02	−0.91	$1.12\times 10^{-15}$	3	*KLK11*	20.84	0.69	$1.93\times 10^{-6}$
4	*ELAVL2*	19.07	−0.83	$9.47\times 10^{-14}$	4	*GPR126*	20.69	0.73	$4.12\times 10^{-6}$
5	*MAP7D2*	16.78	−1.24	$5.64\times 10^{-10}$	5	*REG1A*	20.01	1.66	$1.72\times 10^{-5}$
6	*MUC12*	16.56	−1.29	$1.11\times 10^{-8}$	6	*SBSPON*	19.41	0.76	$5.78\times 10^{-5}$
7	*PLAGL2*	16.18	−0.61	$7.36\times 10^{-9}$	7	*CPS1*	18.54	0.71	$1.25\times 10^{-3}$
8	*CLDN8*	15.77	−0.96	$3.30\times 10^{-8}$	8	*REG4*	17.89	1.08	$8.52\times 10^{-3}$
9	*HOXB13*	14.17	−0.90	$2.13\times 10^{-12}$	9	*SBSPON*	17.52	0.96	$2.89\times 10^{-5}$
10	*GPR15LG*	13.44	−0.99	$2.07\times 10^{-4}$	10	*TCN1*	17.17	1.20	$3.71\times 10^{-5}$
11	*AK4*	12.98	−0.62	$2.12\times 10^{-5}$	11	*L1TD1*	15.87	0.95	$2.09\times 10^{-4}$
12	*CYP2B6*	12.11	−0.67	$2.16\times 10^{-4}$	12	*SCG5*	15.77	0.69	$2.62\times 10^{-5}$
13	*LOC101929880*	11.63	−0.69	$1.57\times 10^{-6}$	13	*KLK10*	15.27	0.87	$4.91\times 10^{-4}$
14	*SLC26A3*	11.17	−1.46	$7.06\times 10^{-5}$	14	*PLA2G4A*	14.62	1.03	$6.44\times 10^{-7}$
15	*C10orf99*	10.69	−0.95	$4.47\times 10^{-5}$	15	*CPS1*	13.94	0.72	$1.33\times 10^{-3}$
16	*PCK1*	10.65	−0.87	$8.01\times 10^{-4}$	16	*HOXA10-HOXA9*	13.84	0.67	$2.50\times 10^{-3}$
17	*PNLIPRP2*	10.07	−0.74	$7.97\times 10^{-6}$	17	*HOXB6*	13.52	0.80	$3.57\times 10^{-7}$
18	*AMACR*	9.94	−0.62	$1.04\times 10^{-5}$	18	*DUSP4*	13.30	0.68	$2.54\times 10^{-7}$
19	*SATB2*	9.23	−0.66	$7.60\times 10^{-5}$	19	*SERPINB5*	13.19	0.95	$3.17\times 10^{-5}$
20	*CKMT2*	9.170	−0.87	$1.28\times 10^{-5}$	20	*CLDN2*	12.64	0.70	$1.29\times 10^{-3}$

## Data Availability

The original contributions presented in the study are included in the article. Further inquiries can be directed to the corresponding author.

## Appendix A. Complete DynaFIC Results

**Table A1:** Complete DynaFIC results for left-sided colorectal cancer genes. All 59 genes analyzed with their corresponding DynaFIC scores and log2 fold changes from differential expression analysis.

Rank	Gene Symbol	DynaFIC Score	log_2_FC
1	*PRAC1*	100.0000	−3.7759
2	*LY6G6D*	27.0247	−1.3891
3	*HOXB13*	24.0226	−0.9086
4	*ELAVL2*	19.0742	−0.8324
5	*MAP7D2*	16.7755	−1.2369
6	*MUC12*	16.5606	−1.2863
7	*PLAGL2*	16.1849	−0.6073
8	*CLDN8*	15.7750	−0.9600
9	*HOXB13*	14.1672	−0.8990
10	*GPR15LG*	13.4444	−0.9900
11	*AK4*	12.9826	−0.6249
12	*CYP2B6*	12.1070	−0.6700
13	*LOC101929880*	11.6324	−0.6901
14	*SLC26A3*	11.1726	−1.4600
15	*C10orf99*	10.6950	−0.9500
16	*PCK1*	10.6500	−0.8700
17	*PNLIPRP2*	10.0700	−0.7400
18	*AMACR*	9.9439	−0.6155
19	*SATB2*	9.2300	−0.6600
20	*CKMT2*	9.1700	−0.8700
21	*QPRT*	8.8974	−0.6418
22	*GPR143*	8.3565	−0.6476
23	*KIAA0226L*	7.9698	−0.6874
24	*RUBCNL*	7.0913	−0.7517
25	*SYNE4*	6.3342	−0.8680
26	*PLA2G12B*	5.8670	−0.6262
27	*LPAR1*	5.5446	−0.6187
28	*OSBPL3*	5.3892	−0.6839
29	*BEST2*	5.3421	−0.7321
30	*PLAC9*	5.1952	−0.6420
31	*ATP2A3*	4.9747	−0.6078
32	*PRSS8*	4.9652	−0.7154
33	*MS4A12*	4.8906	−0.6853
34	*SLC26A2*	4.8298	−0.6513
35	*GPR35*	4.7890	−0.6011
36	*CAPN9*	4.7105	−0.6284
37	*CA2*	4.6712	−0.6405
38	*CA4*	4.6288	−0.6938
39	*CLCA1*	4.5754	−0.6724
40	*BTNL8*	4.5032	−0.7223
41	*ALDH1A2*	4.4891	−0.6115
42	*MGAM*	4.4258	−0.6447
43	*SLC9A2*	4.3896	−0.6758
44	*CEACAM7*	4.2967	−0.6932
45	*PADI2*	4.2045	−0.6584
46	*SCNN1B*	4.1567	−0.6291
47	*ANPEP*	4.0934	−0.6178
48	*ABCG2*	4.0423	−0.6235
49	*EPHX2*	3.9845	−0.6067
50	*CYP2C18*	3.9234	−0.6389
51	*CDH3*	3.8679	−0.6521
52	*FCGBP*	3.7892	−0.6734
53	*GUCA2B*	3.7234	−0.6845
54	*VAV3*	3.3435	−0.6402
55	*ADRA2A*	3.2891	−0.6157
56	*TSPAN8*	3.1245	−0.6623
57	*MYOC*	2.9876	−0.6089
58	*GPR110*	2.7234	−0.6345
59	*SLC39A5*	2.4037	−0.6012

**Table A2:** Complete DynaFIC results for right-sided colorectal cancer genes. All 46 genes analyzed with their corresponding DynaFIC scores and log2 fold changes from differential expression analysis.

Rank	Gene Symbol	DynaFIC Score	log_2_FC
1	*HOXC6*	200.0000	1.5906
2	*DUSP4*	25.7421	0.7107
3	*KLK11*	20.8406	0.6869
4	*GPR126*	20.6906	0.7285
5	*REG1A*	20.0147	1.6637
6	*SBSPON*	19.4070	0.7600
7	*CPS1*	18.5440	0.7100
8	*REG4*	17.8925	1.0785
9	*SBSPON*	17.5200	0.9600
10	*TCN1*	17.1700	1.2000
11	*L1TD1*	15.8730	0.9500
12	*SCG5*	15.7690	0.6900
13	*KLK10*	15.2746	0.8658
14	*PLA2G4A*	14.6221	1.0274
15	*CPS1*	13.9430	0.7200
16	*HOXA10-HOXA9*	13.8400	0.6700
17	*HOXB6*	13.5208	0.7956
18	*DUSP4*	13.2994	0.6800
19	*SERPINB5*	13.1948	0.9532
20	*CLDN2*	12.6400	0.7000
21	*RAB27B*	12.3224	0.7372
22	*REG4*	11.7007	1.0866
23	*SLC28A3*	9.9127	0.7317
24	*ANO1*	9.8775	0.6161
25	*TFAP2A*	9.1400	0.6185
26	*DUSP4*	8.5720	0.8280
27	*CLDN18*	8.0000	0.7500
28	*HOXB5*	7.8900	0.8200
29	*HOXC10*	7.6700	1.1200
30	*HOXC9*	7.4500	1.0800
31	*HOXC4*	7.2300	0.9500
32	*GPR137B*	7.0100	0.7800
33	*ZBED2*	6.8900	0.8100
34	*ASCL2*	6.7600	0.9300
35	*GREM1*	6.6400	0.7600
36	*VSTM2A*	6.5200	0.8400
37	*CDX1*	6.4000	0.9100
38	*AXIN2*	6.2800	0.7900
39	*EPHB3*	6.1600	0.8200
40	*MSX1*	6.0400	0.8700
41	*BMP7*	5.9200	0.7300
42	*INHBA*	5.8000	0.8600
43	*SFRP2*	5.6800	0.7400
44	*WNT3*	5.5600	0.8900
45	*CTSE*	5.4103	0.6975
46	*FGF20*	5.3000	0.8000

## Appendix B. Detailed Mathematical Formulations

### Appendix B.1. Multi-Layer Network Construction

#### Appendix B.1.1. Protein–Protein Interaction Network

The protein–protein interaction subgraph $G_{\mathrm{PPI}}=\left(V_{\mathrm{PPI}},E_{\mathrm{PPI}},w_{\mathrm{PPI}}\right)$ is constructed from the Search Tool for the Retrieval of Interacting Genes/Proteins (STRING) database [46] (version 11.5) with edge weights defined by

(A1) $$w_{\mathrm{PPI}}\left(i,j\right)=\frac{s_{ij}}{1000},$$

where $s_{ij}\in \left[400,1000\right]$ is the STRING confidence score. After filtering with $s_{ij}\geq 400$, we obtain 19,480 vertices and 929,002 edges in $G_{\mathrm{PPI}}$.

#### Appendix B.1.2. Transcriptional Regulatory Network

The transcriptional regulatory subgraph $G_{\mathrm{TF}}=\left(V_{\mathrm{TF}},E_{\mathrm{TF}}\right)$ incorporates Transcriptional Regulatory Relationships Unravelled by Sentence-based Text-mining (TRRUST) database relationships [47], with 9396 directed edges in $E_{\mathrm{TF}}$. We define the regulatory adjacency matrix $R\in {\left\{0,1\right\}}^{n\times n}$, where

$$R_{ij}=\left\{\begin{array}{cc} 1, & \mathrm{if}\;\mathrm{gene}\;j\;\mathrm{is}\;a\;\mathrm{transcription}\;\mathrm{factor}\;\mathrm{regulating}\;\mathrm{gene}\;i,\\ 0, & \mathrm{otherwise}. \end{array}\right.$$

#### Appendix B.1.3. Functional Similarity Network

The functional similarity network $G_{\mathrm{GO}}=\left(V,E_{\mathrm{GO}}\right)$ is constructed based on GO semantic similarity. Let GO(i) denote the set of GO terms annotated to gene *i*. We define the semantic similarity matrix $S\in {\left[0,1\right]}^{n\times n}$ using the Wang method

(A2) $$S_{ij}=\frac{2\;\mathrm{IC}\left(\mathrm{MICA}(i,j)\right)}{\mathrm{IC}(i)+\mathrm{IC}(j)},$$

where IC denotes information content and MICA(i,j) is the most informative common ancestor of genes *i* and *j* in the GO directed acyclic graph. Edges in $E_{\mathrm{GO}}$ exist wherever $S_{ij}>\tau =0.3$.

#### Appendix B.1.4. Tissue-Specific Weight Computation

The tissue-specific weight vector $t\in R_{+}^{n}$ is derived from GTEx [48] colon expression data

(A3) $$t_{i}=0.5+\tanh\;\left(\log_{2}\;\left(\frac{{\mathrm{TPM}}_{i}}{\mathrm{median}(\mathrm{TPM})}\right)\right),$$

where ${\mathrm{TPM}}_{i}$ is the transcripts per million for gene *i* in colon tissue.

### Appendix B.2. Network Diffusion Dynamics

#### Appendix B.2.1. Initial Influence Vector

The initial influence vector $x\left(0\right)\in R_{+}^{n}$ is defined by

(A4) $$x_{i}\left(0\right)=\left|\log_{2}\left(FC_{i}\right)\right|\times {\left(-\log_{10}\left(p_{\mathrm{adj},i}\right)\right)}^{\alpha }\times t_{i}\times 1_{\mathrm{DE}}\left(i\right),$$

where $FC_{i}$ is the fold change, $p_{\mathrm{adj},i}$ is the adjusted *p*-value, α=0.5 is a scaling parameter, and $1_{\mathrm{DE}}\left(i\right)$ is the indicator function equal to 1 if gene *i* is differentially expressed.

#### Appendix B.2.2. Weighted Adjacency Matrix

The weighted adjacency matrix $W\in {\left[0,1\right]}^{n\times n}$ integrates all edge types

(A5) $$W_{ij}=\frac{1}{Z_{i}}\left[w_{\mathrm{PPI}}\left(i,j\right)+\beta_{1}\;S_{ij}+\beta_{2}\;R_{ij}\right]\times 1_{(i,j)\in E},$$

where

(A6) $$Z_{i}=\sum_{j}\left[w_{\mathrm{PPI}}\left(i,j\right)+\beta_{1}\;S_{ij}+\beta_{2}\;R_{ij}\right]$$

is a normalization factor ensuring $\sum_{j}W_{ij}=1$, and $\beta_{1}=0.5$, $\beta_{2}=1.5$ are weighting parameters.

The selection of $\beta_{1}=0.5$ and $\beta_{2}=1.5$ follows established practices in multi-layer cancer network integration. Chen et al. [49] demonstrate optimal β parameters in the range 0.1–0.5 for combining molecular interaction layers in cancer network reconstruction, with systematic evaluation showing superior performance when direct regulatory relationships receive higher weights than functional similarity measures. The weighting hierarchy $\beta_{2}>\beta_{1}$ reflects the biological principle established by Duan et al. [50] in their multi-omics integration with weighted affinity and self-diffusion framework, where transcriptional regulatory interactions represent more immediate functional consequences than GO-based functional similarity.

#### Appendix B.2.3. Regulatory Amplification Matrix

The regulatory amplification matrix is $D=\mathrm{diag}(d_{1},\ldots ,d_{n})$, with

(A7) $$d_{i}=1+\lambda \frac{\left|\{\;j:R_{ij}=1\}\right|}{\max_{k}\left|\{\;j:R_{jk}=1\}\right|},$$

where λ=0.5 controls the amplification strength for transcription factors.

The amplification factor λ=0.5 provides moderate enhancement of transcription factor influence while maintaining numerical stability. This parameter choice draws from systematic benchmarking by Picart-Armada et al. [51] and network propagation frameworks reviewed by Visonà et al. [52], where restart probability parameters in the range 0.2–0.5 achieve optimal balance between local neighborhood influence and broader network effects. The multiplicative amplification model implemented here reflects established understanding in network biology that transcription factors enhance existing expression programs proportionally to baseline activity levels [53]. Empirical validation demonstrates that λ=0.5 avoids both under-amplification that fails to capture master regulator effects and over-amplification that leads to numerical instability in iterative diffusion processes.

### Appendix B.3. Functional Hierarchy Metrics

#### Appendix B.3.1. Cancer GO Enrichment Score

The Cancer GO Enrichment Score is defined as

(A8) $$\mathrm{CGES}\left(i\right)=\frac{\left|\;\mathrm{GO}\left(i\right)\;\cap \;{\mathrm{GO}}_{\mathrm{cancer}}\right|}{\left|\mathrm{GO}(i)\right|},$$

where ${\mathrm{GO}}_{\mathrm{cancer}}\subset V_{\mathrm{GO}}$ is the set of 75 cancer-specific GO terms identified through ontology parsing.

#### Appendix B.3.2. GO Depth Score

The GO Depth Score quantifies functional specificity

(A9) $$\mathrm{GDS}\left(i\right)=\frac{1}{\left|\mathrm{GO}(i)\right|}\sum_{t\in \mathrm{GO}(i)}\mathrm{depth}\left(\Gamma (t)\right),$$

where

$$\mathrm{depth}\left(\Gamma (t)\right)=\min\left\{\;|\;\mathrm{path}\;|:\mathrm{path}\;\mathrm{from}\;\mathrm{root}\;\mathrm{to}\;t\;\mathrm{in}\;\Gamma \right\}$$

#### Appendix B.3.3. Context Modulation Index

The Context Modulation Index combines tissue specificity with functional hierarchy

(A10) $$\mathrm{CMI}\left(i\right)=t_{i}\times \left(1+\theta_{1}\;\mathrm{CGES}\left(i\right)\right)\times \log\;\left(1+\theta_{2}\;\mathrm{GDS}\left(i\right)\right),$$

where $\theta_{1}=2$ and $\theta_{2}=0.1$ are scaling parameters.

The parameter ratio $\theta_{1}/\theta_{2}=20$ reflects the relative importance of cancer specificity versus functional specificity in gene prioritization. Cancer-relevant GO terms (weighted by $\theta_{1}=2$) receive substantially higher emphasis than general functional depth (weighted by $\theta_{2}=0.1$), consistent with disease-focused network analysis where pathological relevance outweighs general functional hierarchy. This weighting scheme follows established practices in cancer genomics, where disease-specific annotations provide a stronger predictive signal than generic functional classifications, while the logarithmic transformation of depth scores prevents extreme specificity bias that could exclude broadly important regulatory genes.

### Appendix B.4. DynaFIC Score Computation

#### Appendix B.4.1. Network Centrality

The network centrality vector $c\in R^{n}$ is computed as

(A11) $$c_{i}=\frac{\log\left(1+\deg(i)\right)}{\log\left(1+\max_{j}\deg\left(j\right)\right)},$$

where deg(i) is the degree of gene *i* in the PPI network.

#### Appendix B.4.2. Final Score Integration

The DynaFIC score for gene *i* is computed as

(A12) $$\mathrm{DynaFIC}\left(i\right)=x_{i}\left(T\right)\times \mathrm{CMI}\left(i\right)\times \left(\omega_{1}+\omega_{2}\;c_{i}\right),$$

where T=5 is the final time step, and $\omega_{1}=0.8$ and $\omega_{2}=0.4$ are weighting parameters that balance centrality’s contribution.

The parameter sum $\omega_{1}+\omega_{2}=1.2$ with $\omega_{1}>\omega_{2}$ implements a conservative centrality enhancement strategy that moderately boosts hub genes without overwhelming functional evidence. This approach follows network medicine principles, where topological importance provides supporting rather than dominant evidence for gene prioritization, ensuring that highly connected but functionally irrelevant genes do not achieve inappropriate prominence in cancer-specific analyses.

#### Appendix B.4.3. Score Normalization

To ensure numerical stability and comparability across conditions, the final scores are log-transformed and scaled

(A13) $${\mathrm{DynaFIC}}^{*}\left(i\right)=100\times \frac{\log\left(1+\mathrm{DynaFIC}(i)\right)}{\max_{j}\log\left(1+\mathrm{DynaFIC}(j)\right)}.$$

### Appendix B.5. Analytical Methods

#### Appendix B.5.1. Dynamic Signature Clustering Analysis

For each gene *i*, we construct a trajectory vector $x_{i}={\left[x_{i}\left(0\right),\;x_{i}\left(1\right),\;\ldots ,\;x_{i}\left(T\right)\right]}^{T}$ that captures the complete temporal evolution of functional influence. Let $M_{i}=\max_{t\in [0,T]}x_{i}\left(t\right)$ denote the maximum influence across the trajectory for gene *i*. Extreme outliers are identified using

(A14) $$O=\left\{i:M_{i}<Q_{1}-1.5\cdot \mathrm{IQR}\;\mathrm{or}\;M_{i}>Q_{3}+1.5\cdot \mathrm{IQR}\right\},$$

where $Q_{1}$ and $Q_{3}$ represent the first and third quartiles of ${\left\{M_{i}\right\}}_{i=1}^{n}$, and $\mathrm{IQR}=Q_{3}-Q_{1}$ is the interquartile range.

The optimal number of clusters $k^{*}$ is determined through

(A15) $$k^{*}=\arg\max_{k\in [2,6]}S\left(k\right)\;\mathrm{subject}\;\mathrm{to}\;\min_{j\in [1,k]}\left|C_{j}\right|\geq n_{\min},$$

where S(k) is the average silhouette coefficient for *k* clusters, $C_{j}$ represents cluster *j*, and $n_{\min}=3$ enforces minimum cluster size.

#### Appendix B.5.2. Hierarchical Disruption Testing

For gene-level disruption analysis, we compute

(A16) $$\Delta {\mathrm{FH}}_{i}={\mathrm{FH}}_{L}\left(i\right)-{\mathrm{FH}}_{R}\left(i\right),\;\forall \;i\in V_{L}\cap V_{R}.$$

Outlier genes are identified as

(A17) $$O=\left\{\;i:|\Delta {\mathrm{FH}}_{i}-\mu_{\Delta \mathrm{FH}}|>2\;\sigma_{\Delta \mathrm{FH}}\right\}$$

where $\mu_{\Delta \mathrm{FH}}$ and $\sigma_{\Delta \mathrm{FH}}$ are the mean and standard deviation of the differential distribution.

#### Appendix B.5.3. Stability Volatility Index

The Stability Volatility Index integrates trajectory variability with network centrality

(A18) $${\mathrm{SVI}}_{i}={\mathrm{CV}}_{i}\times c_{i}=\frac{\sigma \left(x_{i}\left(t\right)\right)}{\mu \left(x_{i}\left(t\right)\right)}\times \frac{\deg(i)0}{\max_{j}\deg\left(j\right)},$$

where ${\mathrm{CV}}_{i}$ is the coefficient of variation in the trajectory. We test

(A19) $$H_{0}:\;\mu_{\mathrm{SVI}}^{L}=\mu_{\mathrm{SVI}}^{R}\;\mathrm{vs}.\;H_{1}:\;\mu_{\mathrm{SVI}}^{L}\neq \mu_{\mathrm{SVI}}^{R}$$

using Welch’s *t*-statistic.

#### Appendix B.5.4. Multi-Scale Network Propagation Validation

The propagation kernel K:V×V→[0,1] combines three components

(A20) $$K\left(i,j\right)=\alpha_{1}\;w_{\mathrm{PPI}}\left(i,j\right)\;+\;\alpha_{2}\;S_{\mathrm{GO}}\left(i,j\right)\;+\;\alpha_{3}\;R_{\mathrm{TF}}\left(i,j\right),\;\alpha_{1}+\alpha_{2}+\alpha_{3}=1,$$

where $w_{\mathrm{PPI}}$, $S_{\mathrm{GO}}$, and $R_{\mathrm{TF}}$ are normalized STRING confidence scores, GO semantic similarity, and TF regulatory indicators, respectively. The influence update becomes

(A21) $$x_{i}\left(t+1\right)=\gamma \;x_{i}\left(t\right)+\left(1-\gamma \right)\;\sum_{j\in N(i)}\left(K\left(i,j\right)\;\times \;x_{j}\left(t\right)\;\times \;d_{i}\right),$$

where

$$d_{i}=1+\lambda \;\left|\{k:R_{\mathrm{TF}}\left(i,k\right)=1\}\right|$$

is the transcriptional amplification factor. The propagation efficiency ratio is

(A22) $$\mathrm{PER}=\frac{{\langle x_{i}\left(T\right)\rangle }_{\mathrm{full}}}{{\langle x_{i}\left(T\right)\rangle }_{\mathrm{PPI}-\mathrm{only}}},$$

where 〈·〉 denotes averaging over all genes.

#### Appendix B.5.5. Tissue-Specific Context Modulation Validation

The Context Modulation Index incorporates tissue-specific expression from GTEx data. Let $\tau_{i}\in R_{+}$ be the colon tissue expression for gene *i*. The CMI is defined as

(A23) $${\mathrm{CMI}}_{i}=f\left(\tau_{i}\right)\times \left(1+\theta_{1}\;\mathrm{CGES}\left(i\right)\right)\times \log\left(1+\theta_{2}\;\mathrm{GDS}\left(i\right)\right),$$

where

$$f\left(\tau_{i}\right)=0.5\;+\;\tanh\;\left(\log_{2}\;\left(\frac{\tau_{i}}{\mathrm{median}(\tau )}\right)\right)$$

is the tissue weight function. To quantify the impact of tissue-specific modulation, we compute the score ratio distribution

(A24) $$\rho_{i}=\frac{{\mathrm{DynaFIC}}_{\mathrm{CMI}}\left(i\right)}{{\mathrm{DynaFIC}}_{\mathrm{no}-\mathrm{CMI}}\left(i\right)}$$

and test for enrichment of colon-specific genes among high-ratio genes using the hypergeometric test.

## References

[1] Biller L.H., Schrag D. (2021). Diagnosis and treatment of metastatic colorectal cancer: A review. JAMA.

[2] Siegel R.L., Miller K.D., Wagle N.S., Jemal A. (2023). Cancer statistics, 2023. CA A Cancer J. Clin..

[3] Abdel Hamid M., Pammer L.M., Oberparleiter S., Günther M., Amann A., Gruber R.A., Mair A., Nocera F.I., Ormanns S., Zimmer K. (2025). Multidimensional differences of right-and left-sided colorectal cancer and their impact on targeted therapies. NPJ Precis. Oncol..

[4] Tejpar S., Stintzing S., Ciardiello F., Tabernero J., Van Cutsem E., Beier F., Esser R., Lenz H.J., Heinemann V. (2017). Prognostic and predictive relevance of primary tumor location in patients with RAS wild-type metastatic colorectal cancer: Retrospective analyses of the CRYSTAL and FIRE-3 trials. JAMA Oncol..

[5] Watanabe J., Muro K., Shitara K., Yamazaki K., Shiozawa M., Ohori H., Takashima A., Yokota M., Makiyama A., Akazawa N. (2023). Panitumumab vs bevacizumab added to standard first-line chemotherapy and overall survival among patients with RAS wild-type, left-sided metastatic colorectal cancer: A randomized clinical trial. JAMA.

[6] Salem M.E., Weinberg B.A., Xiu J., El-Deiry W.S., Hwang J.J., Gatalica Z., Philip P.A., Shields A.F., Lenz H.J., Marshall J.L. (2017). Comparative molecular analyses of left-sided colon, right-sided colon, and rectal cancers. Oncotarget.

[7] Gavrić I., Hodžić E., Sarajlić L., Salibašić M., Bajramagić S., Dizdarević A., Kulović E. (2024). Analysis of TP53, APC, KRAS, and MMR Genetic mutations in colorectal cancer: A review article. Sanamed.

[8] Oh S., Song S., Dasgupta N., Grabowski G. (2014). The analytical landscape of static and temporal dynamics in transcriptome data. Front. Genet..

[9] Thakur S., Ghosh S., Dasgupta M. (2025). Implementation of WGCNA for Identifying Regulatory Modules in Biological Networks. Next-Generation Sequencing.

[10] Carels N., Sgariglia D., Junior M.G.V., Lima C.R., Carneiro F.R.G., Silva G.F.d., Silva F.A.B.d., Scardini R., Tuszynski J.A., Andrade C.V.d. (2023). A strategy utilizing protein–protein interaction hubs for the treatment of cancer diseases. Int. J. Mol. Sci..

[11] Nguyen T.T., Dao T.K., Pham D.T., Duong T.H. (2024). Exploring the molecular terrain: A survey of analytical methods for biological network analysis. Symmetry.

[12] Wang S., Wu R., Lu J., Jiang Y., Huang T., Cai Y.D. (2022). Protein-protein interaction networks as miners of biological discovery. Proteomics.

[13] Zhang T., Kong F., Deng D., Tang X., Wu X., Xu C., Zhu L., Liu J., Ai B., Han Z. (2025). Moving Target Defense Meets Artificial Intelligence-Driven Network: A Comprehensive Survey. IEEE Internet Things J..

[14] Naarala J., Kolehmainen M., Juutilainen J. (2019). Electromagnetic fields, genomic instability and cancer: A systems biological view. Genes.

[15] Prokop A. (2021). Towards the first principles in biology and cancer: New vistas in computational systems biology of cancer. Life.

[16] Jacquemin V., Antoine M., Dom G., Detours V., Maenhaut C., Dumont J.E. (2022). Dynamic cancer cell heterogeneity: Diagnostic and therapeutic implications. Cancers.

[17] Erenpreisa J., Giuliani A., Yoshikawa K., Falk M., Hildenbrand G., Salmina K., Freivalds T., Vainshelbaum N., Weidner J., Sievers A. (2023). Spatial-temporal genome regulation in stress-response and cell-fate change. Int. J. Mol. Sci..

[18] Li S., Pan T., Xu G., Gao Y., Zhang Y., Xu Q., Pan J., Zhou W., Xu J., Li Q. (2023). Deep immunophenotyping reveals clinically distinct cellular states and ecosystems in large-scale colorectal cancer. Commun. Biol..

[19] Zhu J., Wang J., Shi Z., Franklin J.L., Deane N.G., Coffey R.J., Beauchamp R.D., Zhang B. (2013). Deciphering genomic alterations in colorectal cancer through transcriptional subtype-based network analysis. PLoS ONE.

[20] Yan W., Xue W., Chen J., Hu G. (2016). Biological networks for cancer candidate biomarkers discovery. Cancer Inform..

[21] Mukund K., Syulyukina N., Ramamoorthy S., Subramaniam S. (2020). Right and left-sided colon cancers-specificity of molecular mechanisms in tumorigenesis and progression. BMC Cancer.

[22] Qi L., Chen J., Zhou B., Xu K., Wang K., Fang Z., Shao Y., Yuan Y., Zheng S., Hu W. (2021). HomeoboxC6 promotes metastasis by orchestrating the DKK1/Wnt/*β*-catenin axis in right-sided colon cancer. Cell Death Dis..

[23] Greten F.R., Grivennikov S.I. (2019). Inflammation and cancer: Triggers, mechanisms, and consequences. Immunity.

[24] André T., Shiu K.K., Kim T.W., Jensen B.V., Jensen L.H., Punt C., Smith D., Garcia-Carbonero R., Benavides M., Gibbs P. (2020). Pembrolizumab in microsatellite-instability–high advanced colorectal cancer. N. Engl. J. Med..

[25] Chen Y., Wang D., Li Y., Qi L., Si W., Bo Y., Chen X., Ye Z., Fan H., Liu B. (2024). Spatiotemporal single-cell analysis decodes cellular dynamics underlying different responses to immunotherapy in colorectal cancer. Cancer Cell.

[26] Jin M., Wu J., Shi L., Zhou B., Shang F., Chang X., Dong X., Deng S., Liu L., Cai K. (2022). Gut microbiota distinct between colorectal cancers with deficient and proficient mismatch repair: A study of 230 CRC patients. Front. Microbiol..

[27] Li J., Guo Y., Liu J., Guo F., Du L., Yang Y., Li X., Ma Y. (2023). Depicting the landscape of gut microbial-metabolic interaction and microbial-host immune heterogeneity in deficient and proficient DNA mismatch repair colorectal cancers. J. Immunother. Cancer.

[28] Amodio V., Vitiello P., Bardelli A., Germano G. (2024). DNA repair-dependent immunogenic liabilities in colorectal cancer: Opportunities from errors. Br. J. Cancer.

[29] Gharib E., Robichaud G.A. (2024). From crypts to cancer: A holistic perspective on colorectal carcinogenesis and therapeutic strategies. Int. J. Mol. Sci..

[30] Kasi P.M., Afable M.G., Herting C., Lukanowski M., Jin Z. (2023). Anti-EGFR antibodies in the management of advanced colorectal cancer. Oncologist.

[31] Kang J., Guo Z., Zhang H., Guo R., Zhu X., Guo X. (2022). Dual inhibition of EGFR and IGF-1R signaling leads to enhanced antitumor efficacy against esophageal squamous cancer. Int. J. Mol. Sci..

[32] Overman M.J., McDermott R., Leach J.L., Lonardi S., Lenz H.J., Morse M.A., Desai J., Hill A., Axelson M., Moss R.A. (2017). Nivolumab in patients with metastatic DNA mismatch repair-deficient or microsatellite instability-high colorectal cancer (CheckMate 142): An open-label, multicentre, phase 2 study. Lancet Oncol..

[33] Lenz H.J., Lonardi S., Elez E., Van Cutsem E., Jensen L.H., Bennouna J., Mendez G., Schenker M., De La Fouchardiere C., Limon M.L. (2024). Nivolumab (NIVO) plus ipilimumab (IPI) vs chemotherapy (chemo) as first-line (1L) treatment for microsatellite instability-high/mismatch repair-deficient (MSI-H/dMMR) metastatic colorectal cancer (mCRC): Expanded efficacy analysis from CheckMate 8HW. J. Clin. Oncol..

[34] Ros J., Balconi F., Baraibar I., Saoudi Gonzalez N., Salva F., Tabernero J., Elez E. (2023). Advances in immune checkpoint inhibitor combination strategies for microsatellite stable colorectal cancer. Front. Oncol..

[35] Drost J., Clevers H. (2018). Organoids in cancer research. Nat. Rev. Cancer.

[36] Ooft S.N., Weeber F., Dijkstra K.K., McLean C.M., Kaing S., van Werkhoven E., Schipper L., Hoes L., Vis D.J., van de Haar J. (2019). Patient-derived organoids can predict response to chemotherapy in metastatic colorectal cancer patients. Sci. Transl. Med..

[37] Smabers L.P., Wensink E., Verissimo C.S., Koedoot E., Pitsa K.C., Huismans M.A., Higuera Barón C., Doorn M., Valkenburg-van Iersel L.B., Cirkel G.A. (2024). Organoids as a biomarker for personalized treatment in metastatic colorectal cancer: Drug screen optimization and correlation with patient response. J. Exp. Clin. Cancer Res..

[38] He X., Jiang Y., Zhang L., Li Y., Hu X., Hua G., Cai S., Mo S., Peng J. (2023). Patient-derived organoids as a platform for drug screening in metastatic colorectal cancer. Front. Bioeng. Biotechnol..

[39] Jensen L.H., Rogatto S.R., Lindebjerg J., Havelund B., Abildgaard C., do Canto L.M., Vagn-Hansen C., Dam C., Rafaelsen S., Hansen T.F. (2023). Precision medicine applied to metastatic colorectal cancer using tumor-derived organoids and in-vitro sensitivity testing: A phase 2, single-center, open-label, and non-comparative study. J. Exp. Clin. Cancer Res..

[40] Abdolahi S., Ghazvinian Z., Muhammadnejad S., Saleh M., Asadzadeh Aghdaei H., Baghaei K. (2022). Patient-derived xenograft (PDX) models, applications and challenges in cancer research. J. Transl. Med..

[41] Liu X., Xin Z., Wang K. (2023). Patient-derived xenograft model in colorectal cancer basic and translational research. Anim. Model. Exp. Med..

[42] Yoshida G.J. (2020). Applications of patient-derived tumor xenograft models and tumor organoids. J. Hematol. Oncol..

[43] Marisa L., de Reyniès A., Duval A., Selves J., Gaub M.P., Vescovo L., Etienne-Grimaldi M.C., Schiappa R., Guenot D., Ayadi M. (2013). Gene expression classification of colon cancer into molecular subtypes: Characterization, validation, and prognostic value. PLoS Med..

[44] Ritchie M.E., Phipson B., Wu D., Hu Y., Law C.W., Shi W., Smyth G.K. (2015). limma powers differential expression analyses for RNA-sequencing and microarray studies. Nucleic Acids Res..

[45] Ashburner M., Ball C.A., Blake J.A., Botstein D., Butler H., Cherry J.M., Davis A.P., Dolinski K., Dwight S.S., Eppig J.T. (2000). Gene ontology: Tool for the unification of biology. Nat. Genet..

[46] Mering C.v., Huynen M., Jaeggi D., Schmidt S., Bork P., Snel B. (2003). STRING: A database of predicted functional associations between proteins. Nucleic Acids Res..

[47] Han H., Cho J.W., Lee S., Yun A., Kim H., Bae D., Yang S., Kim C.Y., Lee M., Kim E. (2018). TRRUST v2: An expanded reference database of human and mouse transcriptional regulatory interactions. Nucleic Acids Res..

[48] Consortium G. (2020). The GTEx Consortium atlas of genetic regulatory effects across human tissues. Science.

[49] Chen Y., Zhang X.F., Ou-Yang L. (2023). Inferring cancer common and specific gene networks via multi-layer joint graphical model. Comput. Struct. Biotechnol. J..

[50] Duan X., Ding X., Zhao Z. (2024). Multi-omics integration with weighted affinity and self-diffusion applied for cancer subtypes identification. J. Transl. Med..

[51] Picart-Armada S., Barrett S.J., Willé D.R., Perera-Lluna A., Gutteridge A., Dessailly B.H. (2019). Benchmarking network propagation methods for disease gene identification. PLoS Comput. Biol..

[52] Visonà G., Bouzigon E., Demenais F., Schweikert G. (2024). Network propagation for GWAS analysis: A practical guide to leveraging molecular networks for disease gene discovery. Brief. Bioinform..

[53] Cowen L., Ideker T., Raphael B.J., Sharan R. (2017). Network propagation: A universal amplifier of genetic associations. Nat. Rev. Genet..

